# Panoramic visual statistics shape retina-wide organization of receptive fields

**DOI:** 10.1038/s41593-023-01280-0

**Published:** 2023-03-23

**Authors:** Divyansh Gupta, Wiktor Młynarski, Anton Sumser, Olga Symonova, Jan Svatoň, Maximilian Joesch

**Affiliations:** 1 Institute of Science and Technology Austria, Klosterneuburg, Austria; 2 Division of Neuroscience, Faculty of Biology, LMU, Munich, Germany

**Keywords:** Neural circuits, Retina, Neural encoding

## Abstract

Statistics of natural scenes are not uniform—their structure varies dramatically from ground to sky. It remains unknown whether these nonuniformities are reflected in the large-scale organization of the early visual system and what benefits such adaptations would confer. Here, by relying on the efficient coding hypothesis, we predict that changes in the structure of receptive fields across visual space increase the efficiency of sensory coding. Using the mouse (*Mus musculus*) as a model species, we show that receptive fields of retinal ganglion cells change their shape along the dorsoventral retinal axis, with a marked surround asymmetry at the visual horizon, in agreement with our predictions. Our work demonstrates that, according to principles of efficient coding, the panoramic structure of natural scenes is exploited by the retina across space and cell types.

## Abstract (web summary)

The statistics of natural scenes are not uniform—their structure varies dramatically from ground to sky. A combination of theory and experiments revealed that the visual system has adapted to these inhomogeneities to increase coding efficiency.

## Main

The idea that sensory neurons exploit the statistical structure of natural stimuli to minimize the metabolic cost of information transmission has been a guiding principle in neuroscience for over half a century^1–3^. This conceptual framework, known as the efficient coding hypothesis^4^, has provided successful theoretical accounts of sensory coding across species and sensory systems^5–8^ with the retina being the paramount example^9^. Most of the work in the retina has focused on retinal ganglion cells (RGCs), the neurons that relay visual information from the eye to the brain. It has been demonstrated that multiple properties of RGCs—the shape of receptive fields (RFs)^10–13^, organization of RF mosaics^14,15^ and the ratio of ON to OFF RGC cell types^16^—can be explained as adaptations to the natural sensory environment. In all the mentioned cases, ab initio theoretical predictions about efficient encoding of natural scenes have led to a better understanding of the physiological and anatomical properties of the retina.

One way a sensory neuron could implement an efficient code is by removing predictable (or redundant) components from sensory stimuli, in a transformation known as predictive coding (PC). This prominent hypothesis suggests that the center–surround structure of RGC RFs is a manifestation of such design principle^10^. According to this hypothesis, the surround computes a prediction of the stimulus value in the center of the RF. The predicted value is then ‘subtracted’ from the center through inhibition, which dramatically reduces the amount of neural resources used to convey the stimulus downstream. PC and related information–theoretic principles^11–13,17^ typically assume that the structure of natural scenes is uniform across the visual field. However, as demonstrated recently, local contrast and luminance vary prominently across the elevation within the natural visual field of a mouse^18,19^. Such systematic variation affects the signal-to-noise ratio (SNR) of the input to RGCs. To understand how this inhomogeneous noise structure should shape RGC RFs, we developed a simple, predictive coding model. When adapted to natural statistics of mouse vision, our model generates three key predictions linking the shape of optimal RFs and their position within the visual field. First, the relative surround strength should increase with increasing elevation, due to a consistent increase in brightness from the dim ground to the bright sky. Second, the center size should decrease along the same axis. Third, due to a rapid change of signal intensity between lower and upper FOVs, RFs centered on the horizon should have strongly asymmetric surrounds, with the upper half being stronger than the bottom one.

To test these predictions experimentally, we established a new system that enables recording and characterization of the RF structure at high resolution, at the scale of thousands of RGCs in a single retina. Such technological development enabled us to collect a dataset of 31,135 RGC RFs covering the entire central retina, which was crucial to test our theory. We found a close agreement between theoretically optimal RF architecture and the variation of RF shapes across the retina, suggesting that RGCs exploit global asymmetries of natural scenes for maximizing coding efficiency. Furthermore, we explored these adaptations across the diversity of functional RGC types^20^, each thought to share the same physiology, morphology and intraretinal connectivity^21–24^. We identified a systematic dorsoventral variation of the RF shape, regardless of the functional type. Finally, we show that these global adaptations are preserved in awake-behaving animals with intact eyes. Our results thus indicate that adaptations to the panoramic natural statistics structure retinal representations used by the brain.

## Results

### Efficient coding predicts receptive field shapes across the visual field

**Fig. 1:**
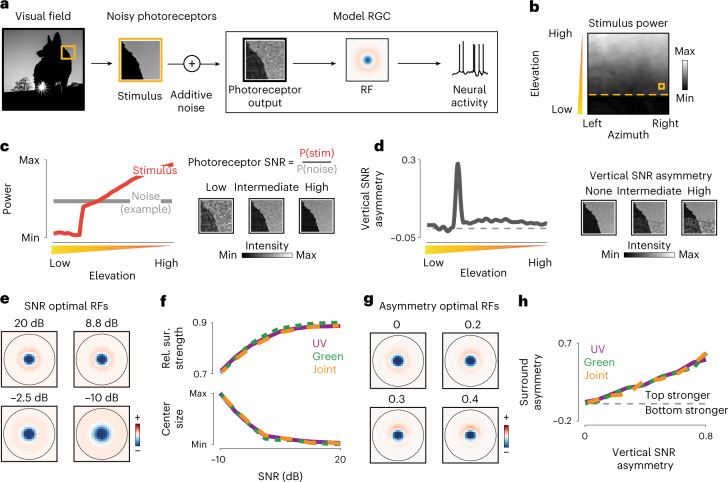
Predictive coding and natural scene statistics. **a**, Schematic of the linear model of a receptive ganglion cell encoding noisy photoreceptor outputs. **b**, Average stimulus power in the mouse FOV in the UV range (natural image data, courtesy of H. Asari^18^). Orange dashed line denotes the simulated horizon. Orange frame illustrates the size of the model RF. **c**, Stimulus power in the UV range (left; red line) and example noise power level (left; gray line) as a function of elevation in the visual field. Increasing stimulus power increases the SNR (right). **d**, Vertical SNR asymmetry in the UV range as a function of elevation in the visual field (left). Change in SNR asymmetry is due to asymmetric power in the stimulus at the horizon line (right). **e**, Predictive coding RFs optimal for different levels of SNR. RFs were smoothed with a 2 × 2-pixel window for display purposes. **f**, Relative surround strength (top) and center size (bottom) of optimal predictive coding RFs increase and decrease respectively, with increasing photoreceptor SNR. Purple, green and orange lines correspond to the UV, green and joint spectra, respectively. **g**, Predictive coding RFs optimal for different levels of vertical SNR asymmetry. RFs were smoothed with a 2×2-pixel window for display purposes. **h**, Surround asymmetry of optimal predictive coding RFs increases with increasing vertical SNR asymmetry of photoreceptor output. Line colors analogous to **f**.

**Extended Data Fig. 1:**
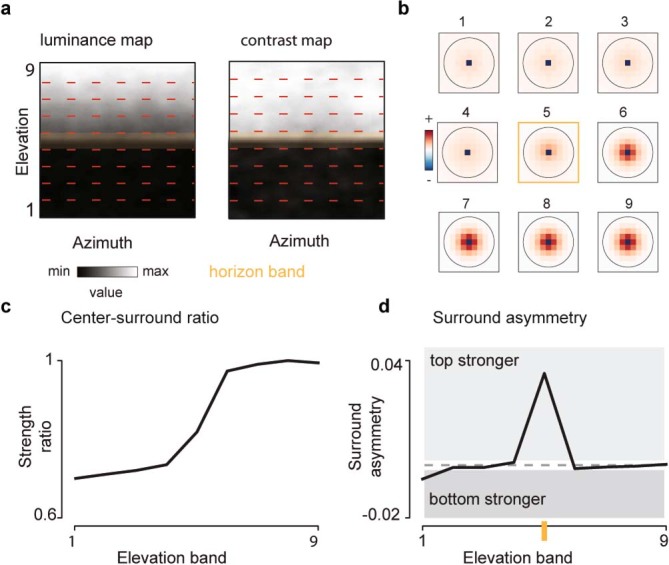
Predictive coding predictions derived from a previously proposed model. (**a**) Average luminance (left panel) and local contrasts (right panel) in the mouse field of view in the ultraviolet range. Red dashed lines separate elevation bands. Orange opaque rectangle denotes the horizon band. Natural image data - courtesy of Hiroki Asari^18^. (**b**) Receptive fields optimized for each band in (a). (**c**) Relative surround-to-center strength as a function of the elevation band. (**d**) Surround asymmetry as a function of elevation band. Orange mark denotes the horizon band.

To understand how the statistical structure of natural scenes shapes RFs across the visual field, we developed a model of sensory coding in RGCs (Fig. 1a). Our approach is closely related to the PC theory, which postulates that RGCs recode outputs of photoreceptor cells to minimize the metabolic cost of sensory information transmission^10^. Following this theory, we modeled neural responses as a linear combination of the RF and natural stimuli, distorted by different sources of constant noise, for example, biochemical or synaptic^9,25,26^ (Fig. 1a). The computation performed by such RFs can be understood as the difference of the weighted center of the stimulus and its surrounding neighborhood. Our model generates predictions consistent with PC (Extended Data Fig. 1) as well as related theories of efficient sensory coding^11,13^.

Predictive coding theory of the retina assumes that statistics of natural stimuli are stationary across the visual field^10^. However, natural scenes are spatially inhomogeneous. To understand this inhomogeneity, we examined a set of natural images collected specifically to study mouse vision^18^. In agreement with previous studies^18,19^, we found that the power of the light intensity decreases gradually with the elevation and drops off suddenly close to the simulated horizon line (Fig. 1b,c). Under the assumption of constant noise level, the SNR of photoreceptor outputs (that is, RGC inputs) should therefore follow the analogous pattern (Fig. 1c). Moreover, due to an abrupt change of the stimulus power, stimuli centered at the horizon yield a highly nonuniform SNR pattern (Fig. 1d). To find how such inhomogeneities could affect sensory representations across the retina, we numerically optimized RFs to minimize the strength of neural responses averaged across a set of natural image stimuli (Methods).

**Extended Data Fig. 2:**
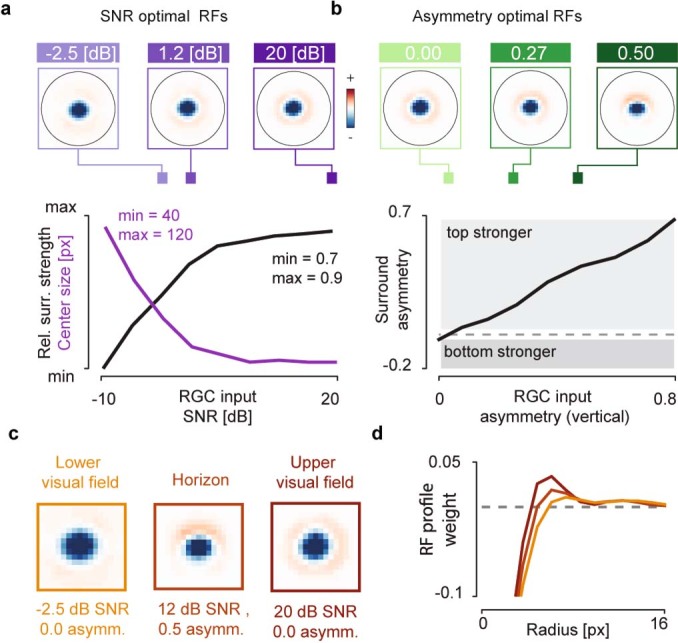
Predictive coding model trained with an alternative set of natural images. We used natural images of animals and landscapes of the African savanna from van Hateren repository, used in^51^ (**a**) Top - RFs optimized at different levels of SNR. Bottom – center size (purple line) and relative surround strength (black line) plotted as a function of the SNR. (**b**) Top - RFs optimized at different levels of the vertical SNR asymmetry. Bottom – vertical surround asymmetry plotted as a function of the vertical SNR asymmetry of the model photoreceptor output (RGC input). (**c**) RFs predicted for different positions within the visual field. (**d**) Horizontal cross-sections of model RFs in (c).

The shape of the optimal RF depends on the relative strength and the structure of noise. When the SNR decreases, the center of the optimal RF broadens, and the surround becomes more diffuse (Fig. 1e). These trends are dependent on the relative change but not on the absolute SNR. This qualitative change is manifested in increasing relative surround strength (Fig. 1f) and decreasing center sizes (Fig. 1f). The optimal RF shape is additionally modulated by the spatial pattern of the SNR (Fig. 1g). When the SNR is spatially nonuniform (for example, when the signal is stronger in the upper half of the stimulus), the optimal RF becomes asymmetric (Fig. 1g). This effect is particularly visible in the increasing asymmetry of the surround as a function of SNR asymmetry (Fig. 1h). Because of such systematic variation in the stimulus power across the visual field, the PC model predicted three qualitative links between the position of a neuron in the retina and the shape of its RF. First, the strength of the RF surround relative to the center should be increasing with elevation across the visual field. Second, the size of the center should increase in the opposite direction. Third, RFs located at the horizon should have surrounds that are substantially stronger in the upper half than in the lower half. Such distribution of RF shapes would indicate that RGCs exploit global statistics of the visual field to maximize the efficiency of sensory coding. These three predictions stand in contrast to the dominant view that RGC RFs are uniform across the retinal surface. Furthermore, predictions of the PC model are reproducible across different ranges of the light spectrum (Fig. 1f,h) and sets of natural stimuli (Extended Data Fig. 1) and depend primarily on weak assumptions about the correlation structure of natural images^10^ (Supplementary Note 1 and Extended Data Fig. 2). We thus consider them to be a robust consequence of the efficient coding hypothesis.

### Large-scale characterization of receptive fields across the retina

**Fig. 2:**
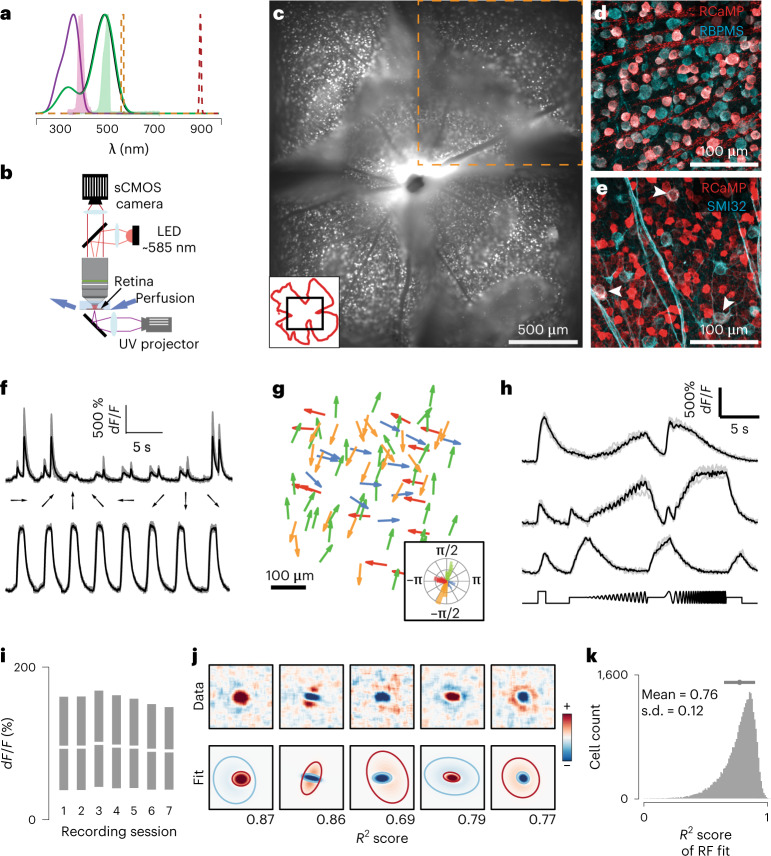
Large-scale retinal receptive field mapping. **a**, Normalized absorption spectra of mouse photoreceptors (purple, S-opsin; green, M-opsin). Normalized emission spectra of the UV and green light emitted by the DLP projector (filled purple, UV; filled green, green stimulus light), epifluorescence (orange) and two-photon (red) excitation are overlaid. **b**, Schematic of the epifluorescence imaging setup. **c**, Montage of five consecutively recorded fields (orange dashed box denotes one field) of a whole-mounted mouse retina from a Vglut2-ires-cre; TITL-R-CaMP1.07-D; ROSA26-ZtTA triple-transgenic mouse. Inset: black, imaged montage; red, retinal outline. **d**, Double-labeled immunostaining of RCamp1.07-expressing RGCs (red) and RBPMS (cyan; *n* = 3). **e**, As in **d** but labeling with SMI32 (cyan). Arrowheads depict double-labeled cells. **f**, Example Ca^2+^ signals (gray, five repetitions; black, mean) from DS (top) and non-DS (bottom) RGCs. **g**, Example distribution of preferred directions in one FOV. Inset shows a polar plot of DS preference. **h**, Example Ca^2+^ signals to chirp stimulus from three different RGCs (gray, five repetitions; black, mean). **i**, Recording Ca^2+^ signal stability across sequentially imaged FOVs for nine retinas (each session lasted ~25 min, 3–7 sessions per retina). White lines denote medians, and minima and maxima of the gray bars indicate the 25th and 75th percentile range of the *dF/F* distribution, respectively. **j**, Example RFs recorded using ‘shifting’ white noise (top) and their respective parametrizations (bottom). Blue and red ellipses correspond to 2 s.d. contours of the ON and OFF Gaussians, respectively. **k**, Histogram of goodness of fit for all recorded RFs.

**Extended Data Fig. 3:**
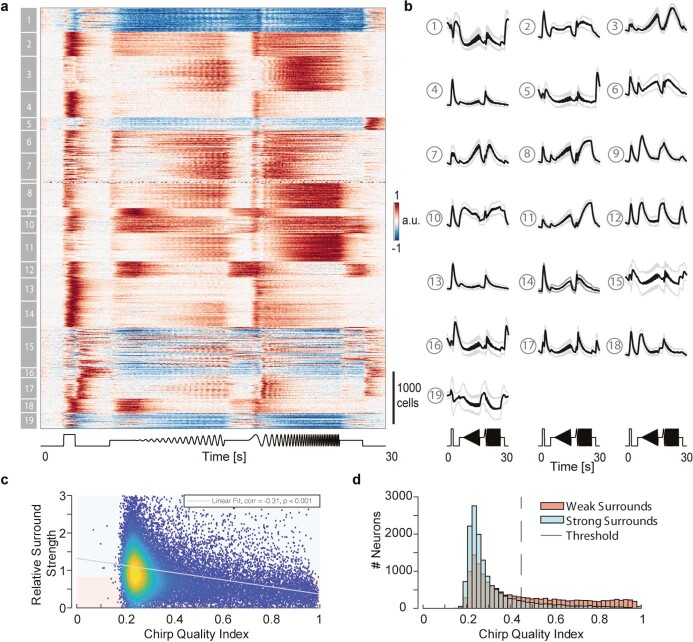
Clustering of Ca^2+^ signals to “chirp” stimulus shows segregation into functional types. (**a**) normalized Ca^2+^ responses to changes in frequency, contrast, and luminance, known as “chirp” stimulus from RGCs, sorted by cluster ids, as determined by GMM. (**b**) Mean and standard deviation of each cluster reveal ON (cluster 2, 4, 8, 9, 10, 11, 13, 14, 18), OFF (5, 12, 15, 16), ON-OFF (3, 6, 7, 17), suppressed-by contrast (1, 19) RGC-types, as well as differences in frequency tuning to slow (for example, cluster 4), mid (for example, cluster 3), fast (for example, cluster 11) and all frequency modulations (for example, cluster 2) or difference in luminance sensitivity (for example, compare ON responsive cluster 14 and 18). Note: clusters 15 and 19 are not homogeneous, as seen in their large standard deviations. (**c**) Chirp-response quality index versus surround strength, determined using their spatiotemporal filters (as in Fig. 2J). Good-quality chirp responses have a strong bias for weak surrounds. (**d**) Distribution of chirp quality index for weak and strong surround, as defined in (c, see color code).

**Extended Data Fig. 4:**
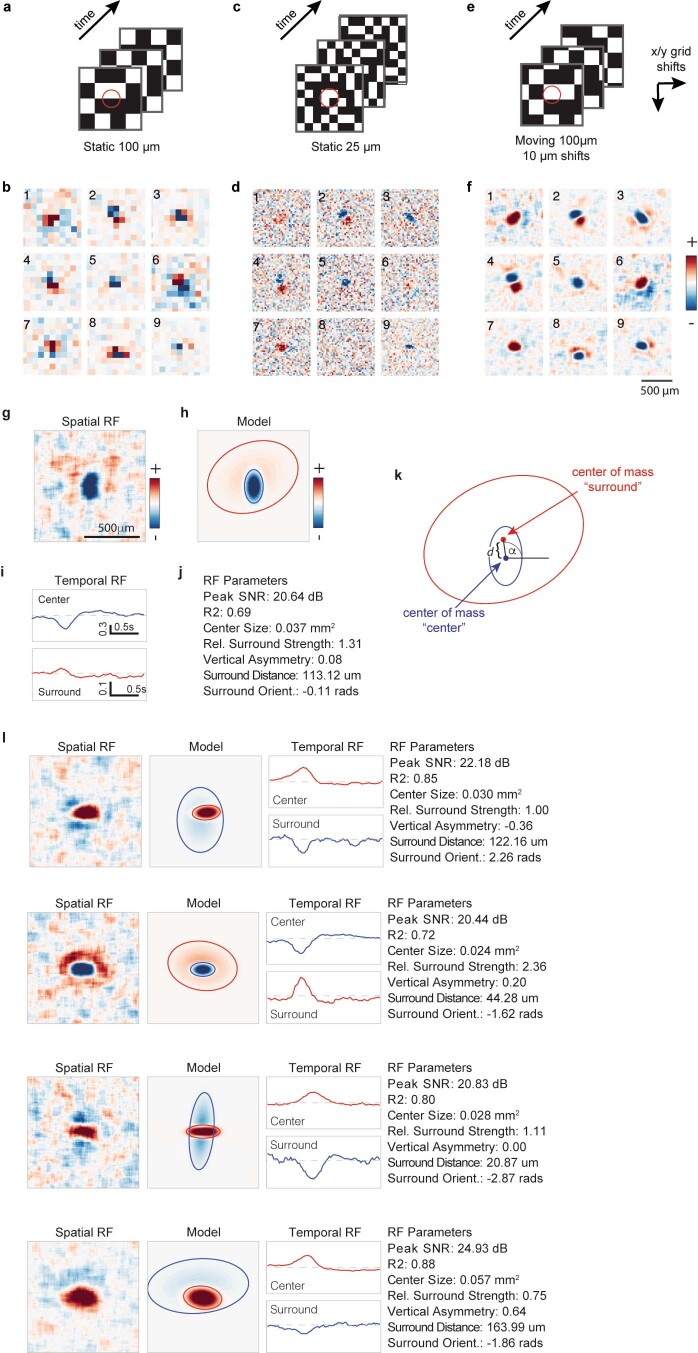
Comparison and parametrizations of RFs. (**a**) Schematic of binary white noise stimuli used for recovering RFs during imaging experiments for static checkers with grid size 100 ×100 μm^2^. (**b**) Spatial RFs of 9 representative neurons were generated from the stimuli above. (**c,d**) Same as (a,b) for the same neurons, but for static 25 μm sized checkers. (**e,f**) Same as in (a,b) for the same neurons but for a shifting checkers with grid size 100 ×100 μm^2^, where the entire grid was shifted by random multiples of 10 μm in both x- and y-axis. While the static 100 μm checkers were too low resolution for automatic analysis and the static 25 μm checkers were unable to drive many neurons strongly enough to elicit sufficient responses for RF reconstruction, the moving white noise stimulus was able to unambiguously recover the most detail in the center–surround structure of RFs. (**g**) Spatial receptive field snapshot at the peak center strength. (**h**) Difference of Gaussians model fitted to the spatial RF. Ellipses represent 2 SD of the two Gaussians. (**i**) Temporal trace of the mean value of pixels within the respective Gaussians (top: center, bottom: surround). Dashed line represents no correlation between stimulus and response. (**j**) Values of different parameters of the RF reconstruction (Peak SNR), the goodness of fit (R2), center-surround structure (Center Size, Rel. Surround Strength) and eccentricity of surround (Vertical Asymmetry, Surround Distance, Surround Orientation). All neurons are plotted with the same scale and limits (after normalization). (**k**) Schematic depicting the parametrization of the receptive field in (g), showing the surround distance (“d”) and orientation (“𝛂”). (**l**) Example parametrizations.

Testing these theoretical predictions requires a high-resolution characterization of RGC RFs from extended regions of the retinal surface. Currently, however, it is not practical to perform such large-scale characterizations with any of the existing methods. Multiphoton imaging approaches can measure large numbers of RGCs^20,27^, but only at a moderate throughput (~150 RGCs at a time^20,27^). Multielectrode array recording approaches have improved this number but are limited by the recording area that is placed on top of the electrode array^15^. Moreover, RF estimates generated by current approaches lack a clear surround structure^20^. To circumvent these limitations, we designed a high-throughput and low-cost epifluorescence approach that enables imaging a larger field of view (FOV; 1.7 mm^2^ sampling at ~1 μm per pixel) and permits >1 h-long recordings of the same FOV while avoiding artifacts caused by small retina wrinkles and laser scanning. Our method takes advantage of red calcium sensors (for example, RCamp1.07)^28^ that separate the Ca^2+^ indicator’s red-shifted excitation light from the opsin absorption spectrum (Fig. 2a,b and Methods), and allows robust responses to ultraviolet (UV) visual stimulation. We used the VGluT2-cre driver line to specifically target RGCs (Fig. 2c), leading to a uniform expression across the entire retina. All RCamp1.07-positive somata correspond to RGCs, as seen by the RGC-specific marker RBPMS^29^ (Fig. 2d). Double-positive cells accounted for ~40% of all RGCs. This expression pattern appears to be RGC-type specific, as seen by co-labeling of SMI32 alpha-RGCs. Alpha-RGCs were consistently excluded from the expression profile, apart from a single, sparse and spatially distributed type (Fig. 2e). Using this line, we were able to reproduce and expand previous large-scale imaging results in single retinas, as seen in direction-selective (DS) and non-DS responses (Fig. 2f), the cardinal DS response distributions (Fig. 2g) and clustering and reproducibility of responses to changes in frequency, contrast and luminance, known as the ‘chirp’ stimulus^20^ (Fig. 2h and Extended Data Fig. 3). By sequentially recording 3–7 FOVs (Fig. 2c), each for approximately 25 min, we could record neural activity from up to ~6 mm^2^ of retinal surface (~40% of the total retinal area). By experimental design, the position of each FOV was random. Moreover, the strength of the functional responses in each consecutive session was unaltered (Fig. 2i). Importantly, using a new ‘shifting’ white-noise approach, where the checker positions are randomly shifted to increase the RF spatial resolution (Extended Data Fig. 4a–f, Methods and ref. ^30^), we were able to estimate high-resolution and high-SNR spatiotemporal RFs for ~85% of recorded cells (Fig. 2j). The quality of these RF estimates allowed for automatic parametrization of the spatial RF into the center and surround using a difference of Gaussians model (Figs. 2j,k, Extended Data Fig. 4g–l and Methods). In total, we recorded 11 retinas, reconstructing and parametrizing 31,135 spatiotemporal RFs, enabling an unprecedented opportunity to index RGC responses across single retinas. This methodology will enable functional developmental screens and circuit dissections due to its simplicity, efficiency and affordability, extending the current retinal research toolbox.

### Receptive fields are adapted to anisotropic natural scene statistics

**Fig. 3:**
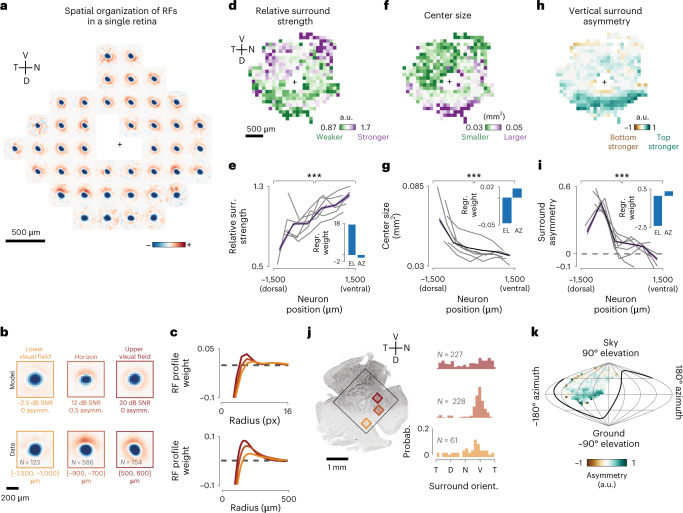
Retina-wide receptive field architecture. **a**, Average spatial RFs of all RGCs pooled from square bins of 300 μm in size at different positions of one retina (*n* = 64 ± 52 cells per bin; black cross indicates the optic nerve head). **b**, Top row, optimal RFs predicted by the model at different elevations of the visual scene. Bottom row, average spatial RFs of neurons along different dorsoventral locations on the retina. **c**, Top, radial profiles of model RFs at different SNR levels. Bottom, mean radial profiles of RGC RFs in bins along the dorsoventral axis. **d**, Mean relative surround strengths of RGCs within 100-μm bins, pooled from *n* = 6 retinas. **e**, Relative surround strengths for RGCs within six equally spaced bins along the dorsoventral axis (color indicates the mean and s.e.m. pooled from *n* = 15,686 RFs, gray lines denote individual retinas, and the inset shows linear regression weights of RF parameter on elevation (EL) and azimuth (AZ)). **f**,**h**, Same as **d**, but for center size and vertical surround asymmetry, respectively. **g**,**i**, Same as **e**, but for center size and vertical surround asymmetry, respectively. **j**, Left, one of the retinas, immunostained for S-opsin. Black box shows the region imaged for RF mapping. Right, normalized histograms of surround orientations of RGCs within corresponding bins marked on the left. **k**, Data from **h** overlaid on a sinusoidal projection of visual space (*n* = 6 retinas). The animal is centered at 0° latitude and 0° longitude facing toward the viewer, and the black line shows the area of the visual field viewed by one eye. *P* values for two-sided Kolmogorov–Smirnov test: 6.11 × 10^−^^5^ (**e**), 2.84 × 10^−^^4^ (**g**) and 1.16 × 10^−^^5^ (**i**); see Extended Data Fig. 6 for extensive statistical comparisons). V, ventral; N, nasal; D, dorsal; T, temporal. a.u., arbitrary units.

**Extended Data Fig. 5:**
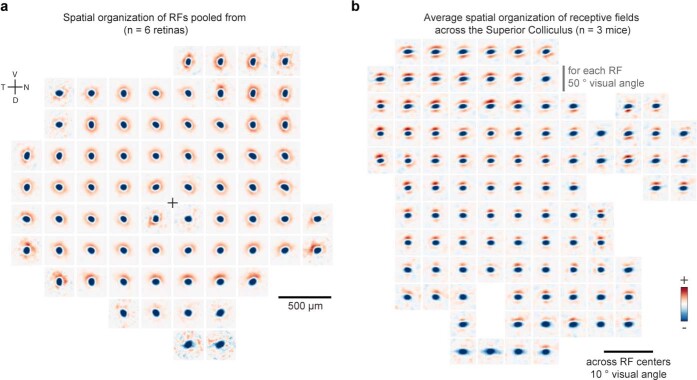
Spatial structure of average RFs across the retina and superior colliculus. (**a**) Average spatial RFs of all RGCs in square bins of size 300 μm at different positions of the retinal surface. As in Fig. 3a, but including cells from 6 retinas (n = 220 ± 200 cells per bin). Black cross: optic nerve head position. (**b**) Average spatial RFs of all RGC boutons in square bins of size 5.6 ° at different positions in visual space. n = 93 ± 68 boutons per bin, only bins with n > = 20 boutons shown.

Taking advantage of the high-resolution RFs, we examined variations in RF shapes and strengths across the retina. Given that the PC model does not determine the polarity of the optimal RF and to globally compare all retinal RFs, ON-center RFs were flipped in sign, such that all centers were negative, and all surrounds were positive. This allowed us to pool across all cells within small bins on the retinal surface and visualize the average spatial RF at different locations of the retina (Fig. 3a and Extended Data Fig. 5a). We observed a general and reproducible trend across 11 retinas: a streak-shaped area where all RF surrounds were oriented toward the optic nerve, and below which, hardly any RF surrounds were observed (Fig. 3a and Extended Data Fig. 5a). To compare these spatial variations of RFs with our theoretical predictions, we oriented six of the recorded retinas to a common coordinate system using the immunohistochemically determined S-opsin gradient. Average RFs in the ventral, centrodorsal and peripheral-dorsal retina (Fig. 3b) qualitatively matched model RFs predicted for the upper, medial and lower visual fields, respectively (Fig. 3b). To confirm the change of relative surround strengths independently from surround asymmetry, we computed the radial profiles of RFs and these also strongly resembled the radial profiles for model RFs (Fig. 3c). Overall, model RFs qualitatively reproduced all aspects of average spatial RFs across different elevations with remarkable detail.

**Extended Data Fig. 6:**
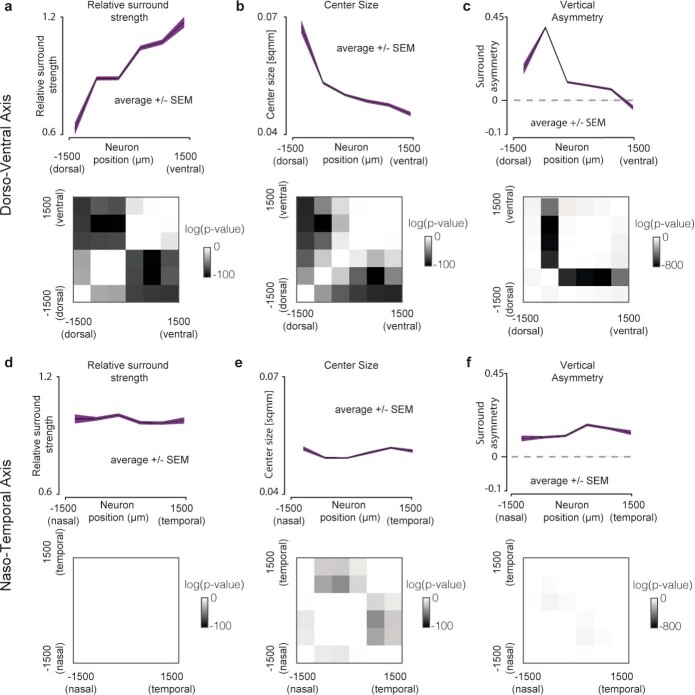
Homogeneity of receptive field architecture in the temporal-nasal axis. (**a**) Top: Mean relative surround strength at 6 different dorsoventral positions. Bottom: p-values of two-sided Kolmogorov-Smirnov tests (with Bonferroni correction) between all pairs of bins. Darker colors represent higher significance levels that the cells in the two corresponding bins have different surround strengths. (**b**) Same as (a), but for trends and significance levels across the naso-temporal axis. (**c,d**) Same as (a,b), respectively, but for center sizes. **(e,f**) Same as (a,b), respectively, but for Vertical surround asymmetry (n = 6 retinas with S-opsin staining).

**Extended Data Fig. 7:**
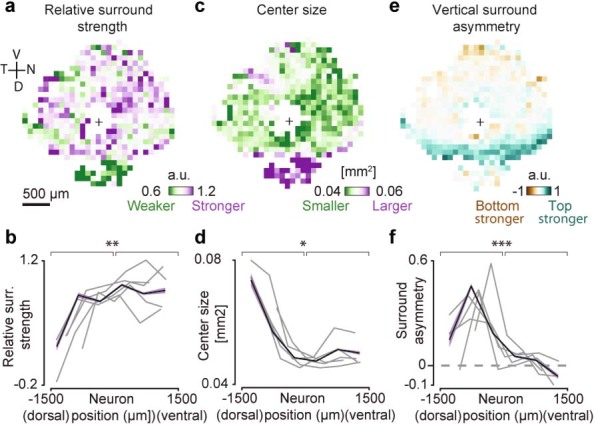
Surround strength, center size and asymmetric streak align in retinas without an S-opsin staining. (**a**) Mean relative surround strengths of RGCs within 100 μm bins, pooled from n = 5 retinas. (Same as Fig. 3d) (**b**) Relative surround strengths for RGCs within 6 equally spaced bins along the presumed dorsoventral axis (color: mean and SEM pooled from n = 15449 RFs, grey lines: individual retinas). (**c** & **e**) Same as (a), but for center size and vertical surround asymmetry, respectively. (**d** & **f**) same as (b), but for center size and vertical surround asymmetry, respectively. (p-values for two-sided Kolmogorov-Smirnov test: (**b**) 0.0047, (**d**): 0.0168, (**f**): 2.3766e-04.

To measure these phenomena quantitatively, we made use of the RF parametrizations and pooled RF parameters for all cells in different two-dimensional (2D; Fig. 3d–h) or one-dimensional (1D; Fig. 3e–i) bins across the retina. In line with our theoretical predictions (Fig. 1f), our analysis shows that the relative surround strength increases gradually along the dorsoventral axis (Fig. 3d), a trend visible in every single retina (Fig. 3e). Next, we explored if we could observe any global change in RF center size. As predicted (Fig. 1f), center sizes decreased only across the dorsoventral axis (Fig. 3f,g and Extended Data Fig. 6). While examining the spatial distribution of differences in upper and lower halves of the RF surrounds, we identified a consistent and prominent asymmetric streak in the dorsal retina, between 700 and 900 μm dorsally from the optic nerve (Fig. 3h,i), as one would expect from asymmetric visual inputs (Fig. 1e). Accordingly, linear regressions weights were substantially stronger in elevation, but not azimuth, for all three trends (Fig. 3e–i and Extended Data Fig. 6). Overlaying the measured RF asymmetries with the opsin gradient indicated that the asymmetry is pronounced in the opsin transition zone (Fig. 3j). To test whether this streak corresponds to the horizon line within the animal’s visual field, we transformed the retinal coordinates to visual coordinates^31^ and used the S-opsin gradient^32^ to define the dorsoventral axis (Fig. 3j,k and Methods). In visual coordinates, the center of this asymmetric streak is located at 0° elevation, spanning the entire azimuth of our imaged FOVs (Fig. 3k), in line with our theoretical predictions (Fig. 1c). Finally, these trends also aligned in five additionally imaged retinas, where the true orientation could not be determined by the opsin gradient (Extended Data Fig. 7).

### Adaptations to natural scene statistics across retinal pathways

**Fig. 4:**
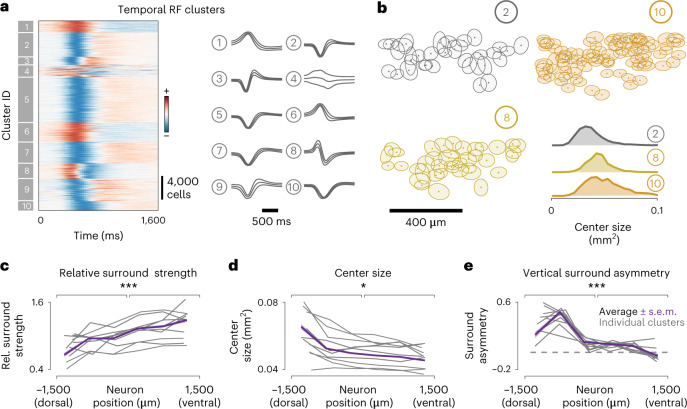
Global adaptation across retinal pathways. **a**, Left, temporal RFs of 31,135 RGCs, grouped by cluster membership. Right, mean and s.d. of temporal RFs in each cluster. **b**, Locations of RGCs (dots) and their RF centers (filled ellipses—1 s.d. of the center Gaussian) belonging to three different clusters in a small region of one retina. Bottom right, distribution of center sizes for these three clusters. **c**–**e**, Trends for relative surround strength (**c**), center size (**d**) and vertical surround asymmetry (**e**) for cells within each cluster (gray) and pooled from all clusters (color), binned as in Fig. 3e. (*P* values for two-sided Kolmogorov–Smirnov test: 6.17 × 10^−^^5^ (**c**), 0.02 (**d**) and 2.62 × 10^−^^7^ (**e**).

**Extended Data Fig. 8:**
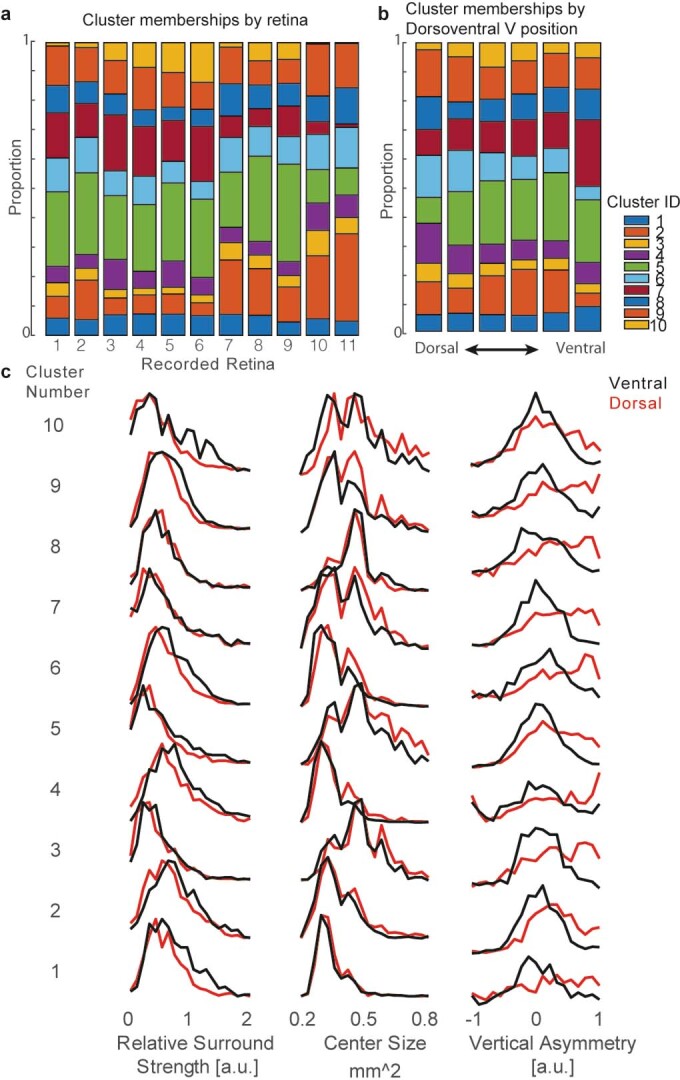
Proportions of cluster membership. (**a**) Fraction of cells from each retina that were classified into each of the 10 temporal RF clusters (from Fig. 4). (**b**) Fraction of cells across dorsoventral positions within bins of width 500 μm. (**c**) Distribution of relative surround to center strength, center sizes and vertical asymmetry for each cluster, subdivided into ventral (above the optic nerve) and dorsal (below the optic nerve). For all statistics, see Supplementary Table 1.

It has long been assumed that specific RGC pathways have stereotyped response properties, shaped by the interactions between direct excitation in their center and indirect inhibition in their surround^33^. Thus, one would expect that these center–surround interactions are uniform across visual space for most RGC pathways. To assess the specificity of the observed RF adaptations across functional RGC pathways, we clustered cells based on the temporal RF profile of 31,135 RGCs into functional groups using a Gaussian mixture model (GMM), as done previously^20^. Consistent with the proportion of RGCs labeled in our line (Fig. 2d), we defined ten functional clusters (Fig. 4a). Chirp responses were not used for clustering because we observed reliable responses only in RGCs with weak surrounds. Therefore, for RGCs with strong surround, the chirps did not help us to match cluster identities (Extended Data Fig. 3c,d), despite finding new response properties that would have aided classification (for example, clusters 3, 11 and 14; Extended Data Fig. 3b). Each cluster group had distinctly shaped temporal filters, corresponding to different functional properties such as ON or OFF selectivity, transient or sustained responses, and monophasic or biphasic selectivity, as seen in their average profiles (Fig. 4a). Cluster membership statistics were conserved across different retinas (Extended Data Fig. 8). Moreover, the relative positions of RGCs belonging to individual clusters tile the retina in a mosaic-like arrangement in many cases (Fig. 4b), confirming that some clusters are indeed functionally distinct and irreducible RGC types^15^. As expected, many clusters represent a combination of RGC types that cannot be identified solely by their temporal profiles (Fig. 4b, cluster 10; see Supplementary Table 1 for tiling statistics). We next used this classification and looked at the relative surround strength, center size and asymmetric strength across clusters (Fig. 4c). As with the global pooling of RF (Fig. 3), cells in each functionally defined cluster increase their relative surround strengths gradually in the dorsoventral axis and decrease their center sizes accordingly. Moreover, all clusters contribute to the asymmetric streak (Extended Data Fig. 8c), consistent with the distribution of asymmetries in the opsin transition zone, where most cells have a strongly oriented surround with a ventral bias (Fig. 3k). All three trends were statistically significant for all clusters in elevation, but not azimuth (Supplementary Table 1). These results indicate that a substantial proportion of RGC pathways adapt to the constraints imposed by natural statistics.

### In vivo panoramic receptive field anisotropies match predictions

**Fig. 5:**
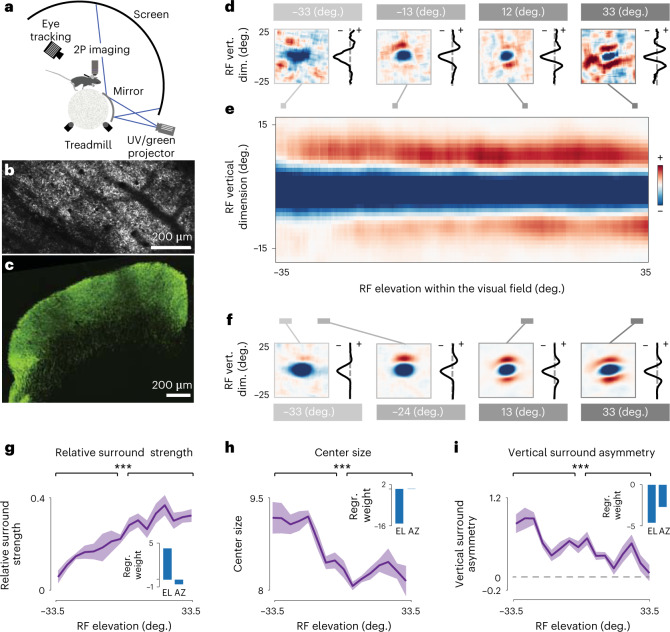
Colliculus-wide retinal ganglion cell’s receptive field architecture. **a**, Schematic of in vivo multiphoton imaging setup. **b**, FOV of a standard multiphoton recording of RGC axons expressing GCaMP8m in the SC (maximum projection, *n* = 10 sessions of three animals). **c**, Immunostaining of GCaMP8m of an example coronal section of the SC (*n* = 3 animals), showing homogeneous RGC labeling across the visual layers (green). **d**, Example RGC bouton RFs recorded using ‘shifting’ white noise (left) and their respective vertical 1D center profiles (1D RFs; right) at different elevation levels (gray lines). Note, ON-center RFs were inverted as done in Fig. 3. **e**, Average 1D RFs in 0.22° bins over elevation (smoothed horizontally in a 5° Gaussian window for display purposes). **f**, Example average RFs binned at a 4.1° visual angle (left), with their respective 1D RFs (right) at different elevation levels (gray bars). **g**, Relative surround strength of 4.1° binned and parametrized average 1D RFs; shading indicates the s.e.m. across azimuth bins (Extended Data Fig. 9g–i). Inset shows linear regression weights of individual bouton (*n* = 9,810) 1D RF parameters on elevation (EL) and azimuth (AZ). **h**,**i**, As in **g**, but for center size and vertical asymmetry, respectively. (*P* values for two-sided Kolmogorov–Smirnov test: 2.91 × 10^−^^10^ (**g**), 1.16 × 10^−^^12^ (**h**) and 1.48 × 10^−6^ (**i**). 2p, two photon.

**Extended Data Fig. 9:**
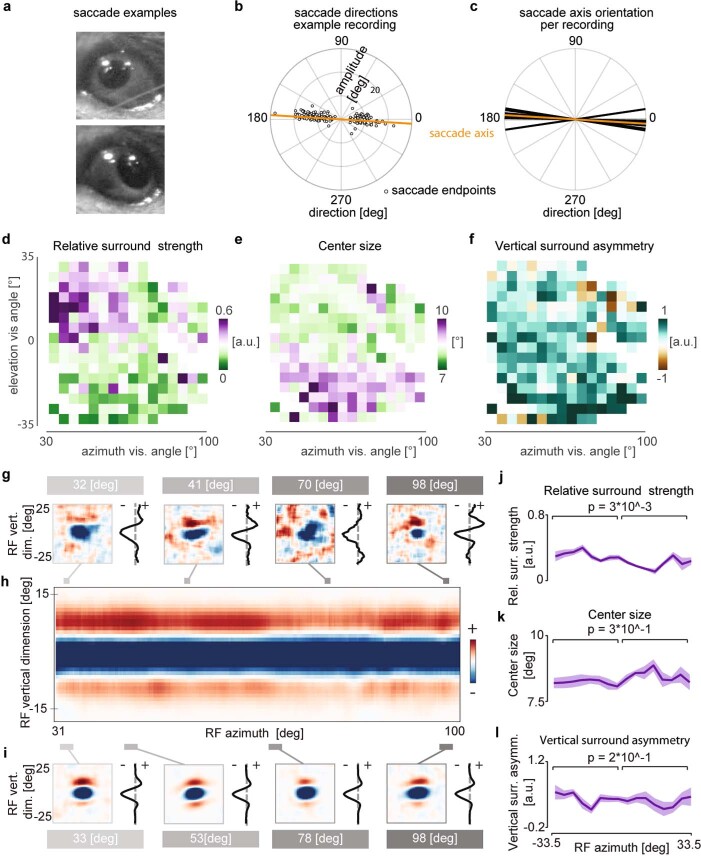
Sampling RGC bouton receptive field architecture across superior colliculus. (**a**) Two example video frame crops of eye in two extreme horizontal positions. (**b**) Polar scatter plot of individual saccade amplitudes and directions (gray dots) in example recording and computed saccade axis (orange line, saccadic orientation tuning r̄ = 0.92). (**c**) Saccade axes of all recordings (n = 10 in 3 mice) close to the horizon (offset = 5.5 ± 2.9 °). (**d**) Relative surround strengths of mean 1-d-RFs within 4.3 ° bins. n = 45 ± 40 boutons per bin, only bins with n > = 5 boutons shown. (**e** & **f**) Same as (d), but for center size and vertical surround asymmetry, respectively. (**g**) Example RGC bouton receptive fields recorded using “shifting” white noise (left) and their respective 1-d RFs (right) at different azimuth positions (gray lines). (**h**) Average 1-d RFs, in smoothed 0.22 ° bins over azimuth. (**i**) Example average receptive fields binned at 2.9 ° visual angle (left), with their respective 1-d RFs (right) at different azimuth positions (gray bars). (**j**) Relative surround strength of 4.1 ° binned average 1-d RFs, shading indicating SEM across elevation bins (shown in (a)). Regression weights for both elevation and azimuth shown in Fig. 5g. (**k, l**) as (j), but for center size and vertical asymmetry, respectively. p-values indicated in j-l for two-sided Kolmogorov-Smirnov test.

To test whether the adaptations to the panoramic visual scene statistics affect sensory coding during behavior in naturalistic conditions, we decided to map RFs of the RGC axonal terminals in the superior colliculus (SC; Fig. 5a,b). These experiments have the advantage of testing our hypothesis in retinas that retain the complete adaptation machinery, from an attached pigment epithelium to functioning pupil constriction. Moreover, they provide important additional insights into the effects of the M-cone and rod pathways, which are saturated in our ex vivo retinal imaging system. For this purpose, we expressed the calcium indicator GCaMP8m^34^ in RGCs, using adeno-associated viruses (AAVs) in three mice. Subsequent implantation of a cranial window above the SC allows for visualization of RGC axonal terminal activity with two-photon calcium imaging in awake, behaving mice. The FOV varied for each recording from 0.32 to 1.85 mm^2^ (median of 0.68 mm^2^) of superficial SC surface (Fig. 5b), encompassing 23 to 57 (median of 41) visual degrees in elevation. GCaMP8m expression spread homogeneously across the SC (Fig. 5c). Using the same previously used ‘shifting checker’ stimulus, we recorded 53,000 terminals in total, reconstructing 10,000 RFs above our quality index (Methods). Compared to the RFs recorded from ex vivo retinas, in vivo measured RFs were blurred along the main axis of saccadic movements (Figs. 2d and 5d; RFs). Thus, to compare across animals, we aligned the RFs of each animal to their respective saccadic plane, which, due to the head fixation, had one consistent axis parallel to the ground to each animal as shown previously^35^ (Extended Data Fig. 9a–c) and corrected the mouse head position to match the retinal coordinates (Methods). To avoid any bias due to eye movements, we then used the 1D profiles (1D RFs) of the orthogonal axis for further analysis (Figs. 5e). By binning and averaging 1D RFs along the lateromedial axis, spanning from the lower visual field to the higher visual field (Fig. 5fg–i and Extended Data Fig. 9d), the three predicted trends became visible: (1) the surround strength increased, (2) the center size reduced and (3) the surround became more symmetric. Similarly to our retinal results (Fig. 3b), the mean 2D RFs qualitatively matched model RFs predicted for the upper, medial and lower visual fields (Fig. 5f). Next, we analyzed the RF parameters on mean 1D RFs across visual space. As with previous results, the RF properties were significantly different above and below the horizon and had substantial regression weights on elevation (Fig. 5g–i) but not on azimuth (Extended Data Fig. 9h–l). Consistently, the average visual maps (Extended Data Fig. 5b) resembled the ones measured ex vivo (Fig. 3d–h). Thus, our in vivo results provide independent corroboration that the visual system is adapted to the constraints imposed by the panoramic natural statistics.

## Discussion

In this study, we leveraged a new, high-throughput neural imaging setup (Fig. 2) to identify a novel kind of adaptation to panoramic scene statistics in the retina. In agreement with theoretical predictions derived from the efficient coding framework (Fig. 1), our experimental results indicate that the global RF architecture is adapted to encode panoramic natural scenes efficiently (Figs. 3 and 4). These results were further corroborated in RGC terminals of awake animals (Fig. 5), indicating that panoramic, efficient representations impact downstream processing during behavior. Classically, RGCs are known to dynamically change the strength of the RF surround in response to varying light levels^36,37^, which is thought to further increase the efficiency of sensory coding^9,38,39^. Our findings demonstrate that, in addition to such dynamic effects, RF shapes are also determined by static factors, namely their position within the visual field. In that way, the retina simultaneously exploits the large-scale spatial and fine-scale temporal structure of the visual space.

How could the visual system establish such global RF architecture without fine-tuning each RGC pathway independently? One partial mechanism would be the nonuniform distribution of spectral sensitivity across the retina^32^. Such distribution has been discussed to be relevant for color vision^40^, contrast coding^19,41^ and the detection of aerial predators in the sky^42^, but simultaneously, could influence the static RF adaptations. For example, whereas the mouse retina has green light-sensing photoreceptors across the entire retina (M-opsin and Rod-opsin), UV sensitivity follows a sharp dorsoventral gradient (S-opsin)^32^. From the RGC’s perspective, both inputs will be added at mesopic conditions, leading to a net enhancement of the UV sensitivity from the ground to the sky. Our in vivo results support this perspective by corroborating the ex vivo findings in an intact eye. Intriguingly, in vivo and ex vivo measured RFs differ subtly. Whereas ex vivo RFs show a clear asymmetrical peak at the horizon, in vivo RFs are more asymmetric across large proportions of the visual field (Fig. 5j). This is consistent with PC predictions because natural image patches located above the horizon tend to be vertically asymmetric (Fig. 1d) due to a gradient of stimulus power (Fig. 1b). Such an RF pattern indicates that other mechanisms, apart from the S-opsin gradient, have to be involved. One possibility could be the circuitry mediating the asymmetric surround of J-RGCs^33,40^, which is ventrally displaced and sensitive to M-cones and rods. Interestingly, the vertical gradient of stimulus power will flatten at lower ambient light levels, for example, at dusk and dawn. In conjunction, the relative strength of UV sensitivity and the antagonistic surround would also decrease^36,37^. In these conditions, efficient coding hypothesis would predict a more homogeneous RF distribution across the dorsoventral axis. Conversely, the horizon will become more prominent in photopic conditions, where rods are less active. In such situations, the in vivo RF architecture should have a localized asymmetric streak, as measured in our ex vivo data. It would be revealing to test if the retina-wide RF organization is dynamically reshaped under scotopic and photopic conditions. Finally, to fully benefit from this panoramic retinal code, the eye should maintain a relatively constant position on the horizon. In agreement with this idea, eye and head movements stabilize the retinal image remarkably well, on average ~10° in azimuthal angle, during behavior^35,43^.

A distinct, yet related question can be asked about the emergence of DS computations in retinorecipient areas, such as the SC, where neurons integrate input from the entire retina, including the asymmetric streak. DS encoding can emerge as a consequence of an asymmetric and time-shifted surround, as shown before^33,44^. Consistent with the measured center–surround asymmetry, some studies have described these neurons as sensitive to upward motion^45^, whereas others do not find such specificity^46,47^. The efficient coding interpretation, such as the one adopted here, suggests that the key to resolving this puzzle might be understanding the statistics of what the animal ought to see in nature.

Our theoretical predictions established qualitative links between properties of RFs and their elevation within the visual field. They can be therefore thought of as a first-order approximation of how the retina is adapted to large-scale, spatial statistics of natural scenes. The exact pattern of global retinal adaptation should vary across species occupying diverse environments. It has been found that dorsoventral opsin gradients are present in many different mammalian species. For example, the rabbit, Chilean subterranean rodent cururo, European mole, the shrew and even the spotted hyena^42,48^ show higher S-opsin expression in the ventral retina. However, not all ecological niches are identical. For example, in dense forests, the vertical gradients of luminance and contrast are less prominent, and a clear horizon line might not be apparent. Interestingly, forest mice species whose opsin distribution has been described present a spatially uniform opsin distribution^49^. This further strengthens our hypothesis, which relates the global organization of the retina to the statistics of the ecological visual field. Understanding this adaptation in more detail will require a careful analysis of stimuli from the ecological sensory niche, as well as an estimation of biophysical parameters such as biological SNR, RF size and tiling to refine our theoretical predictions. The combination of these approaches will be a critical requirement for building a more general theory of vision across the animal kingdom^50^.

## Methods

### Theory

We modeled neural responses *r*_*t*_ as a dot product of the model RF $\overrightarrow \phi$ and noisy stimulus vectors (image patches) ${s}{\overrightarrow{t}}: {rt}={\phi}{\overrightarrow {T}}{st}$, where *T* denotes vector transposition. RFs (filters) were optimized to minimize the following cost function:

$$L\left( {\vec \phi } \right) = \left\langle {\sqrt {r_t^2} } \right\rangle _t + \lambda \mathop {\sum }\limits_{i = 1}^N \phi _i^2d(i)$$

Where *d*(*i*) is the squared distance between the *i*-th value of the RF and the one with the peak absolute value, and *λ* is the strength of the spatial locality constraint. This form of the locality constraint was introduced in ref. ^11^, and it has been demonstrated that it is consistent with RGC properties^11,13^. We note that the activity-related term in the cost function is equivalent to maximizing the sparsity of the neural activity quantified as the average absolute value of neural responses^8^. Without the spatial locality constraint (that is, *λ* = 0), the optimal RF is an oriented, Gabor-like filter. During optimization, to avoid convergence to trivial solutions, the norm of the RF $\overrightarrow \phi$ was constant and equal to 1. Overall, this cost function enforces minimization of activity conveyed downstream, while preserving the information about the image and meeting the locality constraints. Conceptually, this goal is equivalent to that of the PC model^10^. Our model generates predictions consistent with the PC model proposed in ref. ^10^ (Extended Data Fig. 1). It is, however, more flexible, and enables us to capture changes in the center size.

We modeled output of photoreceptor cells (stimuli *s*_*t*_) as natural image patches distorted with the additive Gaussian noise with variance *σ*^2^ that is: $s_{t,i} = x_{t,i} + \xi$, where $\xi \sim N(0,\sigma ^2)$ is the noise term. To simulate different SNR conditions, we manipulated the noise variance level, and optimized RFs for each of the noise levels separately.

We optimized RFs by numerically minimizing the cost function L via gradient descent. For training, we used a dataset of 50,000 square image patches of 27 × 27 pixels in size taken from a dataset of natural images from the mouse visual environment^18^. We sampled images uniformly across the upper and lower visual fields. Each image patch was normalized to have a zero mean and unit variance. To simulate the impact of changing SNR, we normalized images with added noise. During optimization, the dimensionality of natural image data was reduced with principal-component analysis (PCA) to 128 dimensions. For each noise level, dimensionality reduction was performed using the same matrix of PCA components computed on noiseless data. To simulate the impact of changing SNR homogeneity, before normalization we multiplied the bottom half of each image by a scaling factor of less than one, resulting in the range of vertical surround asymmetry values reported in Fig. 1h. After such scaling, we normalized the data and added noise of constant variance. During optimization of RFs on asymmetric stimuli, we computed PCA for each level of SNR asymmetry separately.

In all cases, before optimization, in order to enforce that the RF is centered in the image patch, we initialized RFs with random Gaussian noise with variance equal to 0.1 but set the central pixel value to −1. We independently optimized RFs using images taken in the UV and green parts of the spectrum, as well as in the ‘joint’ spectrum where intensity of each pixel was the average of green and UV values. To ensure that results do not depend on the choice of natural image dataset, we performed the simulations with the images of the African savanna from the van Hateren repository used in ref. ^51^ (Extended Data Fig. 2).

To evaluate RF properties, we defined the size of the RF to be the smallest circle that included 90% of energy (that is, $\phi _i^2$) of optimal RFs averaged across all noise levels (Fig. 1b,c). Within that circle, we defined the center to be all $\overrightarrow \phi$ values smaller than 0, and the surround to be those larger than or equal to 0. The strength of the surround was thus equal to $\mathop {\sum }\limits_{i:\phi _i \ge 0} |\phi _i|$ and the center to $\mathop {\sum }\limits_{i:\phi _i < 0} |\phi _i|$. Sizes of the center were simply numbers of entries that were smaller than 0.

To characterize changes in contrast and luminance across the visual field, we used natural images published in ref. ^18^. We limited our analysis to UV images only; however, light statistics of the green channel do not differ qualitatively. The images provided in ref. ^18^ were divided into two classes—upper visual field and lower visual field. To simulate the visual horizon, we concatenated pairs of images randomly selected from the upper and lower visual fields. We created a dataset of 1,000 such concatenated images and used them to compute the mean and variance of light intensity as estimates of local luminance and contrast, as well as to estimate the SNR as a function of elevation. To estimate the vertical asymmetry of the SNR pattern we used a square stimulus window, and a fixed noise variance. We note that key, qualitative aspects of our predictions do not depend on these choices. For each position *y* of the window along the vertical dimension of the visual field, we computed the vertical SNR asymmetry as: $\mathrm {asym}\left(\, y \right) = \frac{{m_{\mathrm {up}}^{\,y} - m_{\mathrm {down}}^{\,y}}}{{m_{\mathrm {up}}^{\,y} + m_{\mathrm {down}}^{\,y}}}$ where $m_{\mathrm {up}}^{\,y}$ and $m_{\mathrm {down}}^{\,y}$ are sums of the SNR value within the stimulus window above and below its midline, respectively.

### Animals

Mouse protocols were reviewed by the institutional preclinical core facility at IST Austria. All breeding and experimentation were performed under a license approved by the Austrian Federal Ministry of Science and Research in accordance with the Austrian and EU animal laws (BMF-66.018/0017-WF/V/3b/2017). For retinal experiments, triple-transgenic male and female mice (*n* = 8 mice, 3 males, 5 females; *n* = 11 retinas, 5 from the left eye, 6 from the right) aged 5–12 weeks were used for this study (Vglut2-ires-cre (JAX 28863), TITL-R-CaMP1.07-D (JAX 030217) and ROSA26-ZtTA (JAX 012266)). Original strains were obtained from Jackson Laboratories. For in vivo imaging experiments, C57BL/6J (JAX, 000664; *n* = 3, 2 males, 1 female), aged 6–11 weeks at eye injection, were used. The mice were housed in a standard (in vivo inverted) 12-h day–night cycle and euthanized by cervical dislocation before in vitro imaging.

### Statistics and reproducibility

No statistical method was used to predetermine sample size. Low SNR RFs were excluded from analysis, as described below. As done previously^20^, only chirp responses that passed a quality criterion were used for further analysis. Given the nature of the retinal experiments, the location of the recordings was randomized to prevent any biases in the outcome.

### Ex vivo imaging

The dark-adapted mouse retina was isolated under far-red light (LED peak 735 nm, additionally filtered with a 735-nm LP filter eliciting an isomerization rate of ~17 R s^−1^) in oxygenated Ames’ medium (Sigma) with constant bubbling (95% O_2_, 5% CO_2_) at room temperature. Left and right retinas were kept separate for identification. Four incisions were made to flat mount the retina, with ganglion cells facing up, on an 18-mm coverslip (VWR, 631-0153), and held down with a filter paper (Merck, GSWP01300) with a ~2.5 mm × 2.5 mm imaging window cut out. The preparation was then placed in a heated (32 °C) superfusion chamber on the stage of a custom-built upright fluorescence microscope. The retina was left to recover for a minimum of 10 min with the excitation light of the microscope turned on. An amber LED (Thorlabs, M595L4) filtered with a BP filter (Thorlabs, FB580-10) was used for excitation and a BP filter (Thorlabs, 641-75) in series with a 600-nm LP filter (Thorlabs, FEL0600) was used for collection. Background excitation light intensity was at a constant mean photopic intensity of 10^5 ^R s^−1^ per rod (at 585 ± 5 nm). Isomerization rates were determined using opsin templates^52^ and assuming that the mouse rod has an optical density at peak absorption wavelength of 0.015 μm^−1^, a length of 24 μm, a diameter of 1.4 μm and a quantum efficiency of 0.67 (refs. ^53,54^). Each retina was tiled by recording 3–7 different FOVs at ×10 magnification (Olympus XLUMPLFLN20XW objective) using a sCMOS camera (Photometrics Prime 95B) at 10 frames per second and 1.1-μm pixel resolution. Setup was controlled and data were acquired using custom-built LabVIEW software (National Instruments, version 2019).

### Visual stimuli for retinal experiments

Light stimuli were delivered from a modified Texas Instruments DLPLCR4500EVM DLP projector through a custom-made lens system and focused onto the photoreceptors (frame rate of 60 Hz, magnification of 2.5 μm per pixel, total area of 3.2 mm × 2 mm). The projector’s blue LED was replaced with a high-power UV LED (ProLight 1 W UV LED, peak 405 nm), to improve the differential stimulation of S pigments. Two SP filters in series (Thorlabs, FESH0550) were put in the stimulus path to block green light from entering the camera. Intensities and spectra were measured using a calibrated spectrometer (Thorlabs, CCS-100) and a digital power meter (Thorlabs, S130C sensor). A shifting spatiotemporal white-noise stimulus was presented using a binary pseudorandom sequence, in which the two primary lights (green and UV) varied dependently. All white-noise stimuli were presented at a 6-Hz update for 15 min. The checker size was 100 × 100 μm and the entire grid was shifted by random multiples of 10 μm in both *x* axis and *y* axis after every frame. In comparison experiments, static checkers (without shifts) of 100 × 100 μm and 25 × 25 μm were interleaved with the shifting checkers in chunks of 5 min for a total of 20 min for each of the three checker types. A ‘chirp’ stimulus with a 1-s bright step, increasing amplitude (0 to 127 over 8 s) and increasing frequency (0 to 4 Hz over 8 s) was repeated for five trials to reproduce clustering of responses^20^. Moving square gratings (temporal frequency of 0.6 cycles per second and spatial frequency of 0.025 cycles per micron) or a wide bright bar (1 mm s^−1^ speed, 2 mm width) in eight directions, repeated for five trials, were used for assessing direction selectivity. All visual stimuli were generated using the Psychtoolbox (version 3)^55^.

### Histology

After the ex vivo recordings, some of the retinas were fixed with 4% paraformaldehyde (PFA) for 30 min and stained for S-opsin and RFP. Retinas were incubated for 7 d at 4 °C in PBS, containing 5% donkey serum, 0.5% Triton X-100, goat anti S-opsin (1:500 dilution; Rockland, 600-101-MP7) and rabbit anti-RFP (1:1,000 dilution; Rockland, 600-401-379). After washing thrice in PBS for 15 min each, retinas were incubated overnight in secondary antibodies, donkey anti-goat Alexa Fluor 488 (1:1,000 dilution; Abcam, ab150129) and donkey anti-rabbit Alexa Fluor 594 (1:1,000 dilution; Invitrogen, R37119). Retinas were then mounted and imaged with an Olympus VS120 Slidescanner with a ×20 objective. For cell-type characterization, Vglut2-ires-cre; TITL-R-CaMP1.07-D; ROSA26-ZtTA mice were euthanized and perfused intracardially, followed by retina extraction and staining for RBMPS or SMI32, along with RFP (primary antibodies: guinea pig anti-RBPMS (1:500 dilution; Sigma, ABN1376), mouse anti-SMI32 (1:500 dilution; BioLegend, 801701), rabbit anti-RFP (1:1,000 dilution; Rockland, 600-401-379) or mouse anti-RFP (1:500 dilution; MBL, M155-3); secondary antibodies: goat anti-guinea pig Alexa Fluor 647 (1:1,000 dilution; Invitrogen, A21450), donkey anti-mouse Alexa Fluor 647 (1:1,000 dilution; Abcam, A-31571) and donkey anti-rabbit Alexa Fluor 594 (1:1,000 dilution; Invitrogen, R37119). The staining protocol was the same as above and these retinas were imaged with a Leica SP8 confocal microscope.

After the final in vivo recording, mice were terminally anesthetized with ketamine/xylazine (100 mg per kg body weight/10 mg per kg body weight) intraperitoneally (i.p.) and transcardially perfused with PBS, followed by 4% PFA. Brains were extracted and post-fixed in 4% PFA at 4 °C overnight. Brains were then washed and transferred to 30% sucrose solution for cryoprotection overnight at 4 °C and subsequently frozen and the midbrain coronally sliced into 40-µm sections on a Leica SM2010R sliding microtome. Sections were washed and then incubated in PBS, containing 5% donkey serum, 0.3% Triton X-100 and goat anti-GFP (1:2,000 dilution; Abcam, ab6673) overnight at 4 °C. After washing thrice in PBS for 15 min each, brain sections were incubated for 1 h in secondary antibody solution, donkey anti-goat Alexa Fluor 488 (1:1,000 dilution; Abcam, ab150129), washed thrice again in PBS and mounted on slides, where they were stained with DAPI (not shown) and coverslipped with custom-made Mowiol. Brain sections were imaged with a Nikon CSU-W1 spinning disk confocal microscope at ×20 tile stack acquisition. Shading correction was performed on image stacks with BaSiC^56^ in ImageJ and, finally, maximum projection was performed over the whole stack.

### Preprocessing

Regions of interest (ROIs) were detected automatically from raw calcium movies using Suite2p^54^. Fluorescence traces, *F*, were detrended by computing d*F*/*F*, where the 8th percentile in a 20-s sliding window centered around each time point was taken as the baseline fluorescence. Different FOVs from the same retina were stitched together based on coordinates from the stage motors and repeated ROIs in overlapping regions were manually annotated using an open-source tool^57^. Repeated ROIs with the highest score in Suite2p’s built-in classifier were kept for analysis. The deconvolved signal from Suite2p (with tau = 1.0 s) was used for calculating RFs.

The median d*F*/*F* response across trials was taken as the response to the chirp and normalized by dividing by the maximum of the absolute values across time. Quality Index was computed as in ref. ^20^, and only responses with a score > 0.45 were kept for clustering (8,019 of 30,798 neurons). For moving gratings and bar, the mean across trials and maximum across time was taken as the response in any one direction.

### Receptive field mapping

The RF for each neuron was computed as a calcium-triggered average. The spatiotemporal RF at latency *τ*, position (*x*,*y*) for neuron *i* was computed as

$$\mathrm{RF}\left( {i,x,y,\tau } \right) = \mathop {\sum }\limits_{t = 1}^T s\left( {x,y,t - \tau } \right) \cdot r\left( {i,t} \right) - \mathop {\sum }\limits_{t = 1}^T s(x,y,t - \tau ) \cdot \mathop {\sum }\limits_{j = 1}^N r(j,t),$$

where *r*(*i*,*t*) is the deconvolved response of neuron *i* at time *t*, *s*(*x*,*y*,*t*)) is the white-noise stimulus, *T* is the length of the recording and *N* is the total number of neurons in the recording. The second term in this equation subtracts away the residual distribution of the stimulus and the contribution of slow bleaching that is common to all neurons in that recording and leads to RFs that had noticeably less noise. Only the UV channel of the stimulus was used for RF mapping. The latency *τ* was varied in increments of 0.025 s (40 Hz), and the stimulus was interpolated by using the last frame before a particular time, *t-τ*. The RF for each neuron was normalized between −1 and 1 by subtracting the mean value of the RF at latencies *τ* < 0, and dividing by the maximum absolute value of the entire RF.

The location of the center of an RF was estimated by finding the pixel that varied the most across time, $P_{var} = argmax\;Var^t(x,y)$ where $Var^t\left( {x,y} \right)$ is the variance across time for position (*x*,*y*). Each neuron’s RF was cropped within a square window of edge 1 mm centered on this pixel. The SNR of an RF was computed as the peak-to-noise ratio where the power of noise was estimated in regions with distance >0.5 mm from the point *P*_var_. Only RFs with a peak SNR > 15 dB were kept for analysis (31,135 selected RFs of 37,086 recorded neurons). The location of the RF in time was found in a similar way; $T_{var} = argmax\;Var^{xy}(t)$ here, $Var^{xy}\left( t \right)$ is the variance in space.

### Parametrization of receptive fields

We parametrized spatiotemporal properties of the center and surround of the RFs as a sum of two 2D Gaussian distributions (Gaussians) G_1 _+ G_2_. The first Gaussian represents the center of the RF; its amplitude can be either negative or positive corresponding to an OFF or an ON cell. The amplitude of the second Gaussian is required to be of the opposite sign to model the properties of the surround. We computed a spatial representation of the RF, denoted as RF_2D_ as the median of the RF within a small time window around *T*_var_. To reduce noise, we excluded the pixels that were weakly correlated with *P*_var_ across time. The sum of 2D Gaussians G_1_ + G_2_ was then fitted to RF_2D_. Each Gaussian is defined by the amplitude *A*, center $(m^x,m^y)$, width $(\sigma ^x,\sigma ^y)$ and the orientation angle *θ*. We fit only one Gaussian G_1_ to parametrize the location $(m^x,m^y)$, the size and the orientation *θ*_1_ of the center. In the next step, we fit the sum of Gaussians, where we fixed $(m^x,m^y,\sigma ^{x0},\sigma ^{y0},\theta _1)$ parameters of G_1_ while allowing all other parameters to be fitted anew.

Here we differentiated two types of RFs: a RF with a strong center and a weaker surround that largely overlap, and a RF where both center and surround components are strong and well separated. For the first case, we imposed a constraint such that the center of G_2_ is within the distance *d* = 2 min $(\sigma _1^{x0},\sigma _1^{y0})$ from the center $(m_1^x,m_1^y)$. We implemented this constraint as a penalty sigmoid function $P(\mathrm{dist}(G_1,G_2))$ of the distance between the locations of the center and surround components. We added $P(\mathrm{dist}(G_1,G_2))$ to the Gaussian mixture and allowed it to be prohibitively large for dist(*G*_1_, *G*_2_)> *d*. Encoding the constraint in this manner allowed us to remove the bias for the location of the surround on the diagonals of the RF_2D_, which otherwise happens when fitting Gaussian mixture on the square bound-constrained region. There were no constraints for the second type of the RFs where the surround component is strong and more distant from the center. To decide the type of the RF, we found the maximum and minimum points of the RF, and we computed the distance between them and the ratio of their absolute values. If the ratio of the smaller to the bigger values was less than 0.75 or if the distance between the extrema points was less than *d*, then we classified such an RF as the first type, and as the second type otherwise. Experimentally, we found that imposing such a constraint on the location of the second Gaussian leads to a better fitted sum of Gaussians for RFs with largely overlapping center and surround components. All the fitting procedures were implemented using the nonlinear least-squares solver lsqcurvefit in MATLAB.

Using parametrization, we computed various RF characteristics. We found two sets of pixels corresponding to the center and the surround. Center pixels are the pixels within two standard deviations from the center of the *G*_1_. Surround pixels are the pixels within two standard deviations from the center of the *G*_2_ and that are not center pixels. The center size is the number of pixels in the center set, converted to mm^2^ for display. The relative surround strength is the ratio of the absolute value of sum of surround pixels to the absolute value of sum of center pixels. Vertical surround asymmetry is defined as $(u - l)/(u + l)$ where *u* and *l* denote the absolute value of sum of pixels in the upper and lower halves of the surround pixels, respectively. The distance between the center and the surround is the distance between the center of mass (COM) of the center pixels and the COM of the surround pixels. The orientation is the angle between the horizontal axis and the line connecting the two COMs. Radial profiles were computed as the average values of the pixels in RF_2D_ within rings of increasing radii centered on the point *P*_var_. The average value of the center or surround pixels across time was taken to be the RF center or surround temporal dynamics, respectively. The *R*^2^ goodness of fit was computed as

$$R^2 = 1 - \frac{{\mathop {\sum }\nolimits_i \left( {\mathrm{RF}_{2D_i} - M_i} \right)^2}}{{\mathop {\sum }\nolimits_i \left( {\mathrm{RF}_{2D_i} - \overline {\mathrm{RF}_{2D}} } \right)^2}},$$

where *M* = *G*_1_ + *G*_2_ is the RF parametrization. The values of the above RF parameters are reported for a few representative neurons in Extended Data Fig. 4.

### Retina alignment

All functional imaging experiments were performed with randomized retina orientations. For retinas that were co-stained against S-opsin and RCaMP1.07 (*n* = 6 retinas), the direction with the highest density of S-opsin was taken to be the ventral direction^58^. The stitched maximal projection images of the functional imaging experiments were aligned to the RCaMP channel using the retinal vasculature. In each of these retinas, we observed that a streak of asymmetric surrounds was always consistently present across the dorsal retina. Thus, we assumed this to be a reproducible feature, which we then used to manually align the remaining *n* = 5 retinas that did not have an S-opsin staining (and hence no ground truth orientation). To avoid any potential circular arguments, we present the location of neurons from these five retinas only in Extended Data Fig. 7, with a clear indication that the retinal orientation is presumed.

The coordinates of cells from each retina were then translated to make the optic nerve the zero of the coordinate system and rotated such that the positive *y* axis denoted ventral direction. Left retinas were flipped in the nasotemporal axis such that the positive *x* axis denoted nasal direction for all retinas. All spatial RFs were also translated and rotated accordingly. The cartesian retinal coordinates of cells in the stained retinas were converted to spherical visual coordinates using the R package Retistruct, assuming the optical axis of the mouse eye to be oriented at an azimuth of 64° and an elevation of 22° from the long axis of the animal^31^.

### Binning of receptive field properties

For 2D bins, the retinal space from −1,500 μm to 1,500 μm in both nasotemporal and dorsoventral axes was divided into a square grid and all neurons within each bin were collected. Only 2D bins with at least five neurons were analyzed to minimize sampling bias. The spatial RF values of all neurons within each bin of 300 μm in size were averaged and plotted at the location of the bin in Fig. 3a and Extended Data Fig. 6. The RF parameter values of neurons within each bin of size 50 μm were averaged to yield a 2D map of the parameter, and this map was visualized (without smoothing) in Fig. 3d–h. Owing to its area-preserving property, the sinusoidal projection of visual space was binned in the same way as the retinal surface, and the fraction of cells in each bin that had ventrally oriented surrounds were plotted in Fig. 3k.

For 1D analysis, the bins were defined along the dorsoventral or nasotemporal axis based on the range of coordinates in a particular group (by retina (Fig. 3) or cluster (Fig. 4)). The range was divided into six equally spaced bins and the mean parameter value of neurons within each bin was plotted at the coordinate of the center of the bin. As a summary statistic, two-sample Kolmogorov–Smirnov-tests were performed between ventral and dorsal samples of these binned values (*n* = 56 (Fig. 3) and *n* = 60 (Fig. 4) samples). Two-sample Kolmogorov–Smirnov tests (with Bonferroni correction for multiple comparisons) were also performed between raw values of RF parameters for Extended Data Fig. 6. In addition, the weights of linear regressions of RF parameters in elevation (dorsoventral) and azimuth (nasotemporal) orientations are reported.

### Clustering into functional types

The GMM procedure developed in ref. ^20^ was used for clustering temporal RFs and chirp responses, separately. In brief, after normalization, the trace was first reduced in dimension using PCA (10 components for temporal RFs and 20 components for chirp responses) and then GMM models with diagonal covariance matrices were fitted while increasing the number of clusters. The numbers of clusters were identified to be 10 for temporal RFs and 20 for chirp responses based on elbow points in the respective Bayesian information criteria curves. One chirp cluster (*n* = 68 cells) lacked stimulus-evoked responses and was discarded on visual inspection. To assess the degree of overlap between the RFs of neurons belonging to each of the clusters, we defined the tiling index (TI) of the cluster *K* as the area of the union of all RF centers in a cluster, divided by the sum of their individual areas:

$$\mathrm{TI}_K = \frac{{\mathrm{Area}(\mathop { \cup }\nolimits_{i \in K} \mathrm{RF}_i)}}{{\mathop {\sum }\nolimits_{i \in K} \mathrm{Area}(\mathrm{RF}_i)}}$$

The value of this index for each cluster was computed separately for each retina and the mean and s.d. across retinas are reported in Supplementary Table 1.

### Viral eye injections

For viral-mediated gene transfer, 6- to 11-week-old wild-type C57BL/6J mice (JAX, 000664) were anesthetized with ketamine/xylazine by i.p. injection. A 1/2-inch, 30-gauge needle was used to make a small hole in the temporal eye, below the cornea. Then, 1 μl of vitreous fluid was withdrawn and 1 μl of AAV2.7M8-syn-GCaMP8m viral vector solution (at a titer of ~1 × 10^13^ genome copies per ml, ISTA viral facility) was injected into the subretinal space with a Hamilton syringe and a 33-gauge blunt-ended needle.

### Mouse surgery for in vivo imaging

Two to three weeks after viral eye injections, mice were injected with meloxicam (20 mg per kg body weight, subcutaneous (s.c.), 3.125 mg ml^−1^ solution) and dexamethasone (0.2 mg per kg body weight i.p., 0.02 mg ml^−1^ solution). Anesthesia was induced by 2.5% isoflurane in oxygen in an anesthesia chamber. The mouse was subsequently fixed in a stereotaxic device (Kopf) with a constant isoflurane supply at 0.7% to 1.2% in O_2_ and body temperature controlled by a heating pad to 37.5 °C. After the assertion that reflexes subsided, the cranium was exposed and cleaned of periosteum and connective tissue. A circular craniotomy of 4 mm in diameter was drilled above the left SC and from this point onwards, the exposed brain was constantly irrigated with artificial cerebrospinal fluid. The exposed dura mater was removed, and subsequently, the left transverse sinus was sutured twice with 9-0 monofil surgical suture material (B. Braun) and cut between the sutures. Cortical areas covering the left SC were aspirated with a cell culture vacuum pump (Accuris) connected to a blunt needle of 0.5 mm in diameter. A 3-mm circular coverslip was glued (Norland optical adhesives 61) to a thin-walled custom-made conical ring, made from stainless steel. The coverslip ring was inserted into the cavity left by the aspirated cortex, so that the glass was sitting flush on the surface of the SC. Slight pressure was applied with the help of a thinned toothpick, fixed to the stereotaxic arm. The space around the insert was filled with Dura-Gel (Cambridge Neurotech) and the insert was fixed in place with VetBond (3M). After cleaning and drying the surrounding cranium, a multilayer of glues was applied. First, to provide adhesion to the bone, All-in-One Optibond (Kerr) was applied and hardened by blue light (B.A. Optima 10). Second, Charisma Flow (Kulzer) was applied to cover the exposed bone and fix the metal ring in place by also applying blue light. After removal of the fixation toothpick, a custom-designed and manufactured (RPD) headplate, selective laser-sintered from the medical alloy TiAl6V4 (containing a small bath chamber and micro-ridges for repeatable fixation in the setup), was positioned in place and glued to the Charisma on the cranium with Paladur (Kulzer). Mice were given 300 µl of saline and 20 mg per kg body weight meloxicam (s.c.), before removing them from the stereotaxic frame and letting them wake up while keeping them warm on a heating pad. Another dose of 20 mg per kg body weight meloxicam s.c. and 0.2 mg per kg body weight i.p. dexamethasone was further injected 24 h after conclusion of the surgery. After the implantation surgery, animals were allowed to recover for 1 week.

### In vivo visual stimulation and eye movements

Mice were head-fixed while awake using a custom-manufactured clamp, connected to a three-axis motorized stage (8MT167-25LS, Standa). Mice could run freely on a custom-designed spherical treadmill (20-cm diameter). Visual stimuli were projected by a modified LightCrafter (Texas Instruments) at 60 Hz, reflected by a quarter-sphere mirror (Modulor) below the mouse and presented on a custom-made spherical dome (80 cm in diameter) with the mouse’s head at its center. The green and blue LEDs in the projector were replaced by cyan (LZ1-00DB00-0100, Osram) and UV (LZ1-00UB00-01U6, Osram) LEDs respectively. A double bandpass filter (387/480 HD Dualband Filter, Semrock) was positioned in front of the projector to not contaminate the imaging. The reflected red channel of the projector was captured by a transimpedance photo-amplifier (PDA36A2, Thorlabs) and digitized for synchronization. Cyan and UV LED powers were adjusted so that the reflectance on the screen matched the relative excitation of M-cones and S-cones during an overcast day, determined and calibrated using opsin templates^52^ and a spectrometer (CCS-100, Thorlabs). Stimuli were designed and presented with Psychtoolbox (version 3)^55^, running on MATLAB 2020b (MathWorks). Stimulus frames were morphed on the GPU using a customized projection map and an OpenGL shader to counteract the distortions resulting from the spherical mirror and dome. The dome setup allows the presentation of mesopic stimuli from circa 100° on the left to circa 135° on the right in azimuth and from circa 50° below to circa 50° above the equator in elevation.

Visual stimuli were like ex vivo retinal imaging experiments: A shifting spatiotemporal white-noise stimulus was presented using a binary pseudorandom sequence, in which the two primary lights (cyan and UV) varied dependently. All pseudo white-noise stimuli were presented at a 5-Hz update in 5-min episodes, interleaved with different stimuli (for example, gray screen, moving gratings (not shown)) with a total pseudo white-noise duration of 15–60 min (median of 25 min) per recording. The checker size was a visual angle of 8 × 8° and the entire grid was shifted by random multiples of a 0.4° visual angle in both elevation and azimuth axis after every frame. Eye movements of the right eye were recorded with a camera (Basler acA1920-150um, 18–108 mm macro zoom lens (MVL7000, ThorLabs), set at 100 mm, and infrared illumination of 830 nm) via an infrared mirror at 50 frames per second.

### In vivo retinal terminal imaging

Two-photon axonal terminal imaging was performed on a custom-built microscope, controlled by ScanImage (Vidrio Technologies) running on MATLAB 2020b (MathWorks) and a PXI system (National Instruments). The beam from a pulsed Ti:Sapphire laser (Mai-Tai DeepSee, Spectra-Physics) was scanned by a galvanometric-resonant (8 kHz) mirror combination (Cambridge Scientific) and expanded to underfill the back-aperture of the objective (×16 0.8-NA water-immersion, Nikon); 1.9 × 1.9-mm FOV; 30-Hz frame rates. Fast volumetric imaging was acquired with a piezo actuator (P-725.4CA, Physik Instrumente). Emitted light was collected (FF775-Di01, Semrock), split (580 nm long-pass, FF580-FDi01, Semrock), bandpass filtered (green, FF03-525/50; red, FF01-641/75; Semrock), measured (GaAsP photomultiplier tubes, H10770B-40, Hamamatsu), amplified (TIA60, Thorlabs) and digitized (PXIe-7961R NI FlexRIO FPGA, NI 5734 16-bit, National Instruments). The laser wavelength was set between 920 and 950 nm. Average laser output power at the objective ranged from 57 to 101 mW (median of 69 mW)^56^. A FOV of 0.32–1.85 mm^2^ (median of 0.68 mm^2^) was imaged over 3–7 planes (median of 6 planes) with a plane distance of 14–40 µm (median of 25 µm) at a pixel size of 0.6–1.9 µm (median of 1.3 µm) and a volume rate of 4.3–9.5 Hz (median of 5.0 Hz). Each mouse was recorded in 2–4 imaging sessions on different days. In a subset of mice (*n* = 2) in separate imaging sessions, absence of substantial *z*-motion was verified by injecting 40 µl of Texas Red dextran (3000 MW, X 14.3 mg ml^−1^, diluted in saline, Themo Fisher Scientific) s.c. and imaging brightly red labeled blood vessels at 980 nm^59^.

### In vivo eye movement analysis

Behavior videos were analyzed with DeepLabCut^60^, labeling eight points around the pupil. The eight points were then fitted to an ellipse and the ellipse center position transformed to rotational coordinates under the assumption of eyeball radius = 1.5 mm^61^, using custom Python scripts. The median of all eye positions was set to zero azimuth and elevation, that is, all eye coordinates were relative to the median position. The individual horizontal axis, which varied slightly between mice due to differences in the positioning of the head plate, was corrected by leveraging a behavioral feature of head-fixed mice: Saccadic movements are nearly exclusively in one plane^35^. Saccades were extracted by determining events of fast position changes on a median filtered position trace (median filter window of 0.7 s, minimal saccadic speed of 45° per second, minimal saccade amplitude of 3°, minimal saccade interval of 0.25 s). The preferred saccadic orientation and orientation tuning was determined in a similar fashion as that for neuronal visual orientation tuning based on circular variance:

$${\bar{\alpha}} = \frac{1}{2}arg\left[ {\mathop {\sum }\limits_t r_t{{{\mathrm{exp}}}}(2i\alpha _t)} \right]$$

$$\bar r = \frac{1}{{\mathop {\sum }\nolimits_t r_t}}\left| {\mathop {\sum }\limits_t r_t{{{\mathrm{exp}}}}(2i\alpha _t)} \right|$$

with *ᾱ* as the saccadic orientation angle, *r̄* as saccadic orientation tuning, *r*_*t*_ as saccade amplitude and 𝛼_t_ as direction of saccade *t* (Extended Data Fig. 9a–c). Saccade orientation tuning was very high, with mean selectivity = 0.8.

### In vivo axonal terminal analysis

Functional calcium imaging data were first analyzed with suite2p (v0.10.0)^62^ for motion correction and ROI extraction. ROIs were then curated manually based on morphological and activity shape. Further analysis was performed in custom MATLAB R2021a (MathWorks) scripts: *dF/F*_0_ was estimated based on published procedures^63^ by first subtracting neuropil contamination (from suite2p, fluorescence signal of 350 pixels surrounding the ROI, excluding other ROIs) with a factor of 0.5 (estimated from fluorescence of small capillaries as reported previously). From the neuropil-corrected ROI fluorescence, baseline *F*_0_ was defined as the 8th percentile of a moving window of 15 s^64^. *dF/F*_0_ was then calculated by first subtracting and then dividing the fluorescence trace by the median of the same 15-s window^63^. Fluorescence SNR was defined for each neuron by dividing the 99th percentile of the *dF/F* trace (‘signal’) by the standard deviation of its negative values after baseline correction (‘noise’). Only axonal segments with a fluorescence SNR ≥ 5 were included in further analysis. The deconvolved signal from Suite2p (with tau = 0.7 s) was used for calculating RFs. Note that multiple axonal ROIs can originate from the same RGC. Spatiotemporal RF analysis for in vivo retinal terminals was conducted as for ex vivo RGC imaging, but on visual stimuli downsampled to a resolution of a 1° visual angle. The resulting 50 × 50° RFs were contaminated by eye movements and exhibited a lower SNR (as determined by temporal variance of the most varying pixel over the temporal variance of pixels with >50° visual angle distance) than ex vivo soma recordings, requiring further inclusion criteria: SNR > 15 (as in ex vivo data) and peak variance over time located at tau values between −0.1 and 0.6 s. Additionally, the retinotopic projection pattern of RGCs to the SC was utilized by fitting a map from visual coordinates to collicular space. For each recording, RF center azimuth and elevation values were fitted separately to the location of the ROI in the SC using the ‘poly22’ fit option in MATLAB and using only the highest 15% ROIs in SNR and SNR as a fitting weight. Boutons with peak location of the RF deviating by more than a 20° visual angle from its expected location based on the retinotopy fits were removed from further analysis (828 boutons removed).

The main saccadic axis was used to rotate the computed spatial RFs around their respective center and the center positions in visual space as spherical rotation around the approximate eye axis (65° from frontal direction in the horizontal plane). Finally, while freely moving mice hold their head at an approximate pitch angle of 30° downwards^65^, in vivo imaging allowed only for a pitch angle of 10° downwards. To compensate, the center positions of all RFs were spherically rotated 20° downwards around the main pitch axis (90° from frontal direction in horizontal plane). Note that these calculations only allow an estimate of the position of the horizon in free locomotion.

To avoid biasing the analyses by eye movements, RF parametrization was conducted on mean vertical 1D profiles of extracted 50 × 50° 2D RF crops at the peak azimuthal position ± 1°, where ON center bouton RFs were inverted. To extract parameters from 1D RFs, they were fitted with a difference of two Gaussians, initialized with the central peak magnitude (Mpeak) and width (Wpeak). The fitting procedure was then constrained with amplitude_center_ ∈ [M_peak_/2, inf], amplitude_surround_ ∈ [0,inf], location_center_ ∈ [−20, 20]°, location_surround_ ∈ [−25, 25]° (edge of crop), sigma_center_ ∈ [W_peak_/4, inf] and sigma_surround_ ∈ [W_peak_, inf]. Boutons with a center fit location in 1D RFs of more than 5° or with a surround fit location of more than 25° distant from peak estimation based on variance in 2D RFs, were excluded from further analysis (1,609 boutons removed). Extraction of parameters was identical to ex vivo RF parametrization, except center size, where in vivo 2sigma_center_ of the fit was used.

For presenting RF characteristics of RGC axonal boutons in the SC, the centered 1D RFs were binned and averaged in each bin. RF parameters varying over elevation and azimuth are presented as parameters of the fit on the mean 1D RF in the respective bin. Linear regression weights were computed from the parameters and location of each individual bouton.

### Reporting summary

Further information on research design is available in the Nature Portfolio Reporting Summary linked to this article.

## Online content

Any methods, additional references, Nature Portfolio reporting summaries, source data, extended data, supplementary information, acknowledgements, peer review information; details of author contributions and competing interests; and statements of data and code availability are available at 10.1038/s41593-023-01280-0.

## Supplementary information

Supplementary Information Supplementary Note 1

Reporting Summary

Supplementary Table 1 Statistics of cluster analysis for Fig. 4. *P* values: two-sided Kolmogorov–Smirnov test.

## Data Availability

Data used in the analysis can be found at ISTA data repository: 10.15479/AT:ISTA:12370.

## Code availability

Code used to generate the results is available at GitHub: https://github.com/joesch-lab/panoramic-retina/.
