# Supplementary material for: Panoramic visual statistics shape retina-wide organization of receptive fields
Source: Nat Neurosci. 2023 Mar 23;26(4):606–14. doi: 10.1038/s41593-023-01280-0 (PMC10076217; doi:10.1038/s41593-023-01280-0)
Supplement: Supplementary file 1 — Supplementary Note 1 [file 41593_2023_1280_MOESM1_ESM.pdf]

nature neuroscience

Article

https://doi.org/10.1038/s41593-023-01280-0

Panoramic visual statistics shape retina-wide organization of receptive fields

In the format provided by the authors and unedited

Supplementary Information

Supplementary Note 1

Here by following the derivation and assumptions proposed in [10], we present an alternative, analytically-tractable model of predictive coding in retinal receptive fields. The receptive field model in [10] assumes that the center pixel $s_{t,cent}$ of the t-th stimulus $\vec{s_t}$ is subtracted from its linear prediction computed from the surround $\vec{s}_{t,sur}$. Instead of encoding the raw value of the central pixel, the model RGC encodes the difference between this prediction and the center in order to minimize the dynamic range of its output. The optimal prediction weights $\vec{w}$ are optimized to minimize the mean squared error:

$$E(w) = \langle (s_{t,cent} - \vec{w}^T \vec{s}_{t,sur})^2 \rangle_t$$

where T denotes vector transposition.

The optimal vector of surround weights $\vec{w}$ is a solution to the following equation:

$$R_{-c}\vec{w} = \vec{R}_c$$

where $R_{i,j} = \langle s_{t,i} s_{t,j} \rangle_t$ is the spatial autocorrelation of natural images, and i, j index pixels within an image patch, $\vec{R}_c$ is the autocorrelation vector of the center pixel with all other pixels, and R_{-c} is the square correlation matrix of all pixels without the center pixel.

The correlation function $R_{i,j}$ is approximated analytically as:

$$R_{i,j} = M_i M_j + S_i S_j \exp\left[-\frac{d(i,j)}{D}\right], \text{ for } i \neq j \text{ and,}$$

$$R_{i,j} = M_i^2 + S_i^2 + N^2, \text{ for } i = j,$$

Where M_i and S_i are mean and standard deviation of the i-th entry of the image intensity respectively, $d(i, j)$ is the Euclidean, spatial distance between entries labeled i and j, D is a constant controlling the decay of the correlation, and N is the standard deviation of the noise.

The term $N_i N_j$ vanishes for $i \neq j$ because noise is assumed to be uncorrelated.

To approximate the spatial autocorrelation function of natural images as a function of elevation within the visual field, we created a dataset of images with simulated horizon as described in the Methods. We then divided each of these images into uniformly separated horizontal bands. We sampled square image patches within each band. We then computed mean vectors $\vec{M}^y$ and standard deviation vectors $\vec{S}^y$, where the upper index y indicates the elevation band. Individual entries of these vectors corresponded to mean and variance of pixel values within the elevation band y respectively. We assumed constant values of the decay constant D and noise standard deviation N. Ratio of surround-to-center strength and surround asymmetry were computed as described in the Methods. We note that our results do not depend qualitatively on parameter choice and reveal similar trends across a broad range of parameter values.
