# Supplementary material for: Panoramic visual statistics shape retina-wide organization of receptive fields
Source: Nat Neurosci. 2023 Mar 23;26(4):606–14. doi: 10.1038/s41593-023-01280-0 (PMC10076217; doi:10.1038/s41593-023-01280-0)
Supplement: Supplementary file 2 — Reporting Summary [file 41593_2023_1280_MOESM2_ESM.pdf]

nature portfolio

Corresponding author(s): Maximilian Jösch

Last updated by author(s): 18/1/23

Reporting Summary

Nature Portfolio wishes to improve the reproducibility of the work that we publish. This form provides structure for consistency and transparency in reporting. For further information on Nature Portfolio policies, see our Editorial Policies and the Editorial Policy Checklist.

Statistics

For all statistical analyses, confirm that the following items are present in the figure legend, table legend, main text, or Methods section.

n/a	Confirmed	
☐	☒	The exact sample size (*n*) for each experimental group/condition, given as a discrete number and unit of measurement
☐	☒	A statement on whether measurements were taken from distinct samples or whether the same sample was measured repeatedly
☐	☒	The statistical test(s) used AND whether they are one- or two-sided<br>*Only common tests should be described solely by name; describe more complex techniques in the Methods section.*
☒	☐	A description of all covariates tested
☒	☐	A description of any assumptions or corrections, such as tests of normality and adjustment for multiple comparisons
☐	☒	A full description of the statistical parameters including central tendency (e.g. means) or other basic estimates (e.g. regression coefficient) AND variation (e.g. standard deviation) or associated estimates of uncertainty (e.g. confidence intervals)
☐	☒	For null hypothesis testing, the test statistic (e.g. *F*, *t*, *r*) with confidence intervals, effect sizes, degrees of freedom and *P* value noted<br>*Give P values as exact values whenever suitable.*
☒	☐	For Bayesian analysis, information on the choice of priors and Markov chain Monte Carlo settings
☒	☐	For hierarchical and complex designs, identification of the appropriate level for tests and full reporting of outcomes
☐	☒	Estimates of effect sizes (e.g. Cohen's *d*, Pearson's *r*), indicating how they were calculated

Our web collection on statistics for biologists contains articles on many of the points above.

Software and code

Policy information about availability of computer code

Data collection	Custom code using Matlab (2020b) and LabView (2019) was used for setup control. Psychtoolbox (Version 3) was used for the generation of visual stimuli.
Data analysis	ROIs were detected automatically using Suite2p, Pachitariu et al. 2017 (bioRxiv). Follow up analysis was performed using custom code programmed in Matlab. Cartesian retinal coordinates were converted to hemispherical coordinates using Retistruct, Sterratt et al. 2013 (Plos Comput. Biol.). Code used to generate the results is available at Github: https://github.com/joesch-lab/panoramic-retina

For manuscripts utilizing custom algorithms or software that are central to the research but not yet described in published literature, software must be made available to editors and reviewers. We strongly encourage code deposition in a community repository (e.g. GitHub). See the Nature Portfolio guidelines for submitting code & software for further information.

Data

Policy information about availability of data

All manuscripts must include a data availability statement. This statement should provide the following information, where applicable:

- Accession codes, unique identifiers, or web links for publicly available datasets
- A description of any restrictions on data availability
- For clinical datasets or third party data, please ensure that the statement adheres to our policy

Data used in the analysis is available at IST DataRep: https://doi.org/10.15479/AT:ISTA:12370

Field-specific reporting

Please select the one below that is the best fit for your research. If you are not sure, read the appropriate sections before making your selection.

☒ Life sciences ☐ Behavioural & social sciences ☐ Ecological, evolutionary & environmental sciences

For a reference copy of the document with all sections, see nature.com/documents/nr-reporting-summary-flat.pdf

Life sciences study design

All studies must disclose on these points even when the disclosure is negative.

Sample size	No sample size calculations were used for this study. Our data size is larger than most other studies (largest retina RF data-set) and the number of independent measurements matches the standard of the field.
Data exclusions	Only retinas that had full coverage were used (n=9). All cells that had high SNR receptive fields were used (85% of total)
Replication	11 independent retinas show same global trends from 8 animals (3 male, 5 female). 3 independent superior colliculi were imaged and all attempts of replication were successful (2 male, 1 female).
Randomization	The location of imaging FOV was randomized as well as the orientation of the retinas. For the in vivo experiments, one large FOV was required per session, thus, consecutive imaging biases would not have been a problem.
Blinding	Experimenter was blinded to the orientation of the retinas to avoid any imaging biases. Orientations were determined post-hoc through the Sopsin gradient in 6 retinas. In 5 additional retinas, the S-opsin gradient could not be determined. We used this for a supplementary figure to , show the three trends are also aligned in these independent experiments. For in vivo experiments, RF were defined by their response properties after recordings.

Reporting for specific materials, systems and methods

We require information from authors about some types of materials, experimental systems and methods used in many studies. Here, indicate whether each material, system or method listed is relevant to your study. If you are not sure if a list item applies to your research, read the appropriate section before selecting a response.

Materials & experimental systems

n/a	Involved in the study	
☐	☒	Antibodies
☒	☐	Eukaryotic cell lines
☒	☐	Palaeontology and archaeology
☐	☒	Animals and other organisms
☒	☐	Human research participants
☒	☐	Clinical data
☒	☐	Dual use research of concern

Methods

n/a	Involved in the study	
☒	☐	ChIP-seq
☒	☐	Flow cytometry
☒	☐	MRI-based neuroimaging

Antibodies

Antibodies used	Primary Antibodies:<br>goat anti S-opsin (Rockland 600-101-MP7)<br>guinea pig anti-RBPMS (Sigma ABN1376)<br>mouse anti-SMI32 (BioLegend 801701)<br>rabbit anti-RFP (Rockland 600-401-379)<br>mouse anti-RFP (MBL M155-3)<br><br>Secondary Antibodies:<br>goat anti-Guinea pig Alexa Fluor 647 (Invitrogen A21450)<br>donkey anti mouse Alexa Fluor 647 (Abcam A-31571)<br>donkey anti-rabbit Alexa Fluor 594 (Invitrogen R37119)<br>donkey anti-goat Alexa Fluor 488 (Abcam ab150129)
Validation	All primary antibodies are commonly used in the field and have been validated previously. E.g., Reinhard et al. 2019 (Elife), Bleckert et al. 2014 (Curr. Biol.), Rodriguez et al 2014 (J Comp Neurol,) Simons et al. 2021 (Neurobiology of Disease).

Animals and other organisms

Policy information about studies involving animals; ARRIVE guidelines recommended for reporting animal research

Laboratory animals	Triple transgenic female and male C57/Bl6 mice, aged 5-12 weeks, were used in this study. Genotypes: Vglut2-ires-cre (JAX 28863), TITL-R-CaMP1.07-D (JAX 030217) and ROSA26-ZtTA (JAX 561 012266). Mice were housed at 60 % humidity and room temperature (approx. 21 C)
Wild animals	No wild animals were used.
Field-collected samples	No field collected samples were used.
Ethics oversight	All breeding and experimentation were performed under a license approved by the Austrian Federal Ministry of Science and Research in accordance with the Austrian and EU animal laws (Animal protocol: BMF-66.018/0017-WF/V/3b/2017).

Note that full information on the approval of the study protocol must also be provided in the manuscript.
